# Quarterly Periscope of Practical Medicine: Part I

**Published:** 1827-04

**Authors:** 


### Quarterly Periscope

OF

### PRACTICAL MEDICINE;

BEING

### The Spirit of the Medical Journals,

*Foreign and Domestic;*

WITH COMMENTARIES.

### PART I.

"Ore trahit quodcunque potest, atque addit acervo."

### 1. ON INTERMITTENT IRRITATIONS. BY M. DUFAU.*

THE disciples of Broussais have very ingeniously availed themselves of the phenomena of irritation, to shew that inflammation itself may be of an intermittent character, and thus to support the doctrine of ague being, like all other fevers, dependent on local inflammation, the latter of a periodical type. Without being entire converts to the doctrine of either Broussais or Clutterbuck respecting fever, we may allow, what, indeed, common observation shews, that irritation, with or without the phenomena of phlogosis, very often assumes a periodical character; and on this account we shall dedicate a few pages to the paper of M. Dufau, which contains many interesting cases.

He first (and very properly) tells us what he means by the term irritation. Our organs answer to the impressions made on them by external agents, in virtue of a property which we term *excitability.* When this excitability is in a due and healthy degree of exercise, we call it *excitation;* but when raised beyond that point by a too powerful stimulation, it constitutes *irritation.* This we consider to be a simple and correct definition of irritation.

This state presents different phenomena, according to the organic element in which it predominates. Confined to the nervous structure, it causes *pain,* more or less acute, and then receives the name of neurosis, or, more properly, neuralgia. When the irritation extends to the sanguiferous capillaries, and is accompanied by the phenomena of heat, redness, and swelling, we call it *inflammation.* It takes the denomination of *hæmorrhagic* irritation, if the sanguiferous capillaries give way, and pour out their contents. Finally, it is termed *lymphatic* irritation, or *sub-inflammation,* when it predominates in the lymphatic series of

* Journal Général de Médecine.

vessels, and is accompanied by an afflux of serous fluids to the part, with slight or no pain, and without redness or heat.

"Observe," says our author, "attentively an external part of the body when invaded by inflammation. Pain is the first symptom—then redness, heat, and swelling succeed. What are we to infer from this train of phenomena? Why, that the stimulant, or exciting cause, first acted on the nervous system of the part, and afterwards invaded the other textures, causing a fluxionary movement to that point. The hæmorrhagic irritation is only a modification of the sanguineous irritation."

This distinction between irritation confined to the nervous structur and that which is extended to the sanguiferous vessels, is founded in observation and confirmed by practice. The treatment of nervous irritation is very different from that which we employ in the sanguineous—and the lymphatic irritation requires a treatment different from both the others. These three modes of irritation present different phenomena, according to the organs or structures in which the irritation is seated. After this slight exposé, our author proceeds to the facts by which he illustrates the subject of *intermittent irritation.*

*Case* 1. M. D. became affected, in January, 1825, with a violent pain in the right temple, which recurred every second day. The pain was very circumscribed, and was greatly exasperated by any noise, and even by the impression of light. Neither heat, redness, or swelling of the part could be perceived, nor was there the slightest febrile movement in the system. The paroxysms lasted five or six hours, and terminated by a copious perspiration from the head. In the intervals, the health was excellent. Twelve grains of sulphate of quinine, administered in a lavement, completely stopped the paroxysms. This may be considered a specimen of nervous intermittent irritation, characterised by pain alone, and without any fluxionary movement in the vessels.

*Case* 2. A gentleman of acute nervous susceptibility, and very subject to irritation of the stomach and bowels, was seized, in the night of the 16th August, with violent colicky pains, and vomiting and purging of glairy mucous matters, mixed with some blood. These symptoms continuing the next morning, fifteen leeches were applied to the anus, and the patient confined to very rigid regimen. About mid-day the symptoms ceased. 18th. He remained well, and took food. In the night of the 18th, the symptoms returned, as on the night of the 16th, accompanied by burning heat about the anus. The pulse was slightly accelerated—the tongue natural. The return of the symptoms was attributed to the food he had taken, and twenty leeches were applied to the anus. Food was prohibited. All the symptoms ceased at 12 o'clock on the 19th. From this time till midnight of the 20th he was perfectly well, but abstained rigorously from all solid food. At midnight, the symptoms returned as violent as ever, and now M. Dufau recognized a tertian colitis, the pain occupying the region of the transverse colon in particular. This time, fifteen leeches were applied, and no subsequent paroxysm ensued.

It may be remarked, that this complaint occurred at a time when dysentery prevailed in the town where M. Dufau resided, (Mont de Marsan,) and therefore he could not consider the disease as any thing more than a modification of dysentery—that is, an intermittent dysentery. He offers it as an example of periodical colitis—or intermittent inflammation of the mucous membrane of the colon.

*Case* 3. Our author was called to a labouring man on the 13th of August, who had been ill six days, but of whose history he could obtain little information. The patient was free from fever, but had a continual call to stool, voiding only glairy mucus mixed with blood. He ordered a rice ptisan, a dose of cream of tartar—and at night a slight opiate, with aromatics. Next morning our author found the patient quite well, and complimented himself on the success of his prescription. But in the evening of the 15th he was sent for in great haste, as his patient was reported to be dying. He found him scarcely alive, being nearly insensible, his countenance cadaverous, his extremities cold and covered with frigid perspiration, the pulse scarcely perceptible, and the patient passing much black blood, with constant straining at stool. An opiate was administered, and directions given to commence the cinchona as soon as the symptoms were mitigated. The paroxysm terminated in a few hours—and an ounce and a half of bark was exhibited, at short intervals. No paroxysm returned, and the patient continued well.

Our author concludes from the symptoms, that the disease was dysentery of the intermittent type, and the cure by cinchona, he thinks, confirms this conclusion.

*Case* 4. *Periodical Peritonitis.* A young man, 24 years of age, was carried to the Hospital of Mont de Marsan, on the 4th of April, 1824, and found by our author, presenting the following symptoms:—acute pain over the whole abdomen, which was hot, tense, distended, (météorisé,) and exceedingly tender on pressure. He frequently vomited yellow and bitter matters. The tongue was moist, and of natural colour—thirst moderate—head-ache—countenance shrunk—breathing quick—skin burning hot and dry—pulse hard, sharp, and quick. The complaint had commenced the preceding evening, and was ushered in by a rigor. Our author recognized in these phenomena a case of peritonitis. Fourteen ounces of blood were taken from the arm—starvation enjoined—diluent drinks—emollient fomentations—lavements. At the evening visit, our author was not a little surprised to find his patient completely free from every one of the symptoms above-described. They had disappeared on the occurrence of a copious perspiration. The next day (5th April) the patient continued free from complaint, and some very light aliment was permitted. In the morning visit of the 6th, our author found his patient labouring under precisely the same symptoms as those described on the 4th, when he entered the hospital, and ushered in, as before, by a strong rigor. M. Dufau was at a loss to know whether he had an intermittent inflammation, or a relapse of continued peritonitis to treat. He applied 25 leeches to the abdomen. In the evening, a complete intermission took place, after a copious perspiration. 7th. The patient was in good health, and craved for food, which was granted to him. In the night of this day, the symptoms returned, but in so mitigated a form, that our author did not deem it necessary to prescribe either depletion or the bark. The paroxysms did not return till the 20th day, when, after some excesses in diet, the phenomena first described occurred. Only abstinence and fomentations to the abdomen were ordered. The succeeding day (28th April) was apyrectic, but on the 29th a most tremendous paroxysm came on, attended with all the symptoms of violent peritonitis. Wishing to determine whether the bark would be as successful in stopping the paroxysms now, as depletion and starvation had been before, M. Dufau prescribed the sulphate of quinine. No paroxysm afterwards occurred, and the patient was discharged from the hospital cured.

M. Dufau remarks that this case requires no comment. But we would just observe, that it does not appear quite determined whether the local symptoms of peritonitis were the cause of the general disturbance of the system, or merely an accompaniment. We have seen many cases where the hot stage of an intermittent presented phenomena in the head, the chest, and the abdomen, bearing all the marks of inflammation in those parts for the time being, and vanishing at the termination of the paroxysm. But these *local* phenomena we attributed to the general excitement taking a run, as it were, upon some organ or part that had been more predisposed to disorder than other organs and parts. We may have been wrong, however, and it is for more minute investigation to determine whether these local inflammations (if they be really inflammations, and not mere irritations, or excessive degrees of excitement) are the causes, the accompaniments, or the consequences of the general pyrectic commotion in the system. We are much more anxious to discover the truth than to support any doctrine to which we may have been attached by early prejudice or subsequent observation.

*Case* 5. J. Bouillet, aged four years, of feeble constitution, large head, and precocious intellect, became dull, pallid, and drowsy, on the 20th May, 1823. Some slight convulsive movements were observed in the muscles of the face on that day, but the pulse was not disturbed, nor the temperature of the skin increased. Our author conceived that, in these symptoms, he recognized a slight *cerebral irritation*, which had not yet excited any sympathetic affections of other systems or organs. A pediluvium was advised, and, in the evening, the child appeared quite well. The night was good, but towards 12 o'clock next day, the boy became suddenly pale, cold, and complained of lancinating pains at the crown of the head, succeeded by inclination to vomit. After this, he suddenly became insensible, and his eyes and face were agitated with convulsive movements. The face got very red—the head excessively hot, as also the surface of the body—the pulse could not be properly estimated. These symptoms are often attributed to the irritation of worms in the intestines, and M. Dufau does not contend that this is not very frequently the case; but he rightly believes that, wherever the irritation may commence, it has affected the brain sympathetically, when the symptoms above-mentioned have taken place. In the present case, he looked upon the cerebral irritation as primitive or idiopathic, from the largeness of head, precocity of intellect, nervous susceptibility, and absence of all signs of abdominal affection. Fifteen leeches were therefore applied to the temples—cold applications were made to the head—and a warm bath to the inferior half of the body. Under these means the convulsive motions ceased—the boy recovered his senses, but continued to complain of pain in the top of the head—skin hot—pulse hard and very quick—and there was still some subsultus tendinum. Towards the evening an abundant perspiration broke out, and all the febrile and other phenomena quickly vanished. After an interval of health, the same symptoms again returned, and there was no longer any doubt that the disease was of a periodical character. It was put down by our author as "*intermittent arachnitis.*" The sulphate of quinine was now administered, and the disease returned no more.

When we recollect that the *arachnitis* of our author means an *irritation* extended from the nerves to the vessels of the part—that this irritation is intermittent—and that it is cured by tonics, we need not entertain so much repugnance to the term "*intermittent arachnitis.*" The fact is, that it is—it must be, different in its nature, from common or continued inflammation, because it is attended by different phenomena, and requires a different treatment. In short, it bears the same analogy to common inflammation that an ague does to a continued fever.

*Case* 6. Augusta Lestage, aged 26 months, with large head, and florid complexion, was seized on the 1st of May, 1823, with shivering, succeeded by intense heat, thirst, &c. The tongue was red—nausea, vomiting, diarrhœa—abdominal distention. Leeches to the abdomen and fomentations, our author having recognized an inflammatory irritation of the digestive tube and peritoneum. The leeches were not applied as he had directed; but a copious perspiration having taken place in the evening, the symptoms vanished. 2d May. Passed without any fever. 3d May. An accession of fever with the same symptoms as before described. In two hours after the accession, a new order of phenomena was developed. The little patient suddenly became insensible, and all the muscles of the body were agitated with convulsions. The face became highly coloured—the neck red and swelled—mouth ejecting foam, &c. Our author now saw that there was an extension of the inflammatory irritation to the brain. Fourteen large leeches were applied to the epigastrium, and afterwards the lower half of the body was put into the warm bath, the head being kept cool by icy applications. The symptoms soon subsided, an abundant perspiration having broken out. 4th May. Passed without any bad symptom. 5th. A new accession of the fever, the cerebral affection being still more intense than before. The same means were employed as on the 3d, with the addition of leeches behind the ears. At six o'clock in the afternoon an intermission took place. Convinced that the antiphlogistic measures were not sufficient to quell the disease, the sulphate of quinine was administered during the succeeding apyrexia, and no more accessions took place.

*Case* 7. Celestine Caupenne, aged six years, was seized in the night of August 24th, with alarming symptoms, and our author was summoned. He found the little girl insensible, and violently convulsed—head burning hot—countenance greatly flushed—eyes fixed—skin arid—pulse hard and quick. She had gone to bed in perfect health. Our author could not but consider these symptoms as the signals of cerebro-spinal irritation. The child had been exposed on the preceding day to an intense sun, and there was no indication of any gastric affection. Twelve leeches were applied to the base of the skull, while cold lotions were kept on the top of the head, and warm fomentations to the feet. The leeches fell off; but the convulsions continued. The little patient was therefore put into the warm bath, a small stream of cold water was poured on the head. The convulsions gradually subsided. The child was carried to bed, and a perspiration broke out. The fever ceased entirely. The 25th was passed in good health ; but at 2 o'clock the next morning she was seized with the same symptoms as before, and equally intense. The same means were repeated, and the same termination of the paroxysm occurred. The intermittent character of the disease being now unequivocally established, the bark was given in lavements, and the paroxysms stopped.

Our author could bring forward a great number of cases of intermittent gastritis and enteritis, of various types, cured during the year 1823, with great facility, by one or two applications of leeches—occasionally a general bleeding, and strict antiphlogistic regimen. He ought to have added, however, what indeed the foregoing cases shew, that the quinine still more certainly stopped the paroxysms than the depletion. At the same time we cannot but approve the plan of previous local bleeding, even in these intermittent irritations, where one particular organ or texture is more threatened than others.

Dr. Dufau observes, that he was one of the earliest disciples of Broussais, and still adheres to that part of his master's doctrine which assigns a local inflammation as the cause of what are called idiopathic or essential fevers. But he, for a long time, relinquished Broussais' doctrine respecting intermittent fevers, not being able to form an idea of an "*intermittent inflammatory irritation.*" A more close observation of facts has induced him to alter his opinions, and come to the conclusion that idiopathic and intermittent fevers are essentially of the same nature —"produced often by the same causes—cured often by the same means—and occasionally convertible into each other." Why the same causes should at one time produce a continued, and at another, an intermittent fever, our author acknowledges his ignorance. Be it so; but we find fault with M. Dufau, for saying these fevers are cured by the same means. On the contrary, they generally require different modes of treatment—shewing, in our humble opinion, that the *intermittent* inflammatory irritation is different in its nature from *continued* inflammation. Our author, however, diverges so far from the doctrine of his master, as to allow that the *irritation* is not always in the stomach and bowels, though very often so. It may be in the brain, chest, or organs of the abdomen. But the fact is, that, in a majority of intermittent, and still more of continued fevers, the inflammatory irritation is so equally divided, during the paroxysm, among the great organs of the body, that it is utterly impossible to say which is the particular, or even the principal seat of the irritation. And when it does manifest itself more in one organ than another, we have no certain proof that it is the *cause* of the paroxysm in one class, or the continued excitement in the other class of fevers. There is just as much reason to conclude that its predominance in a particular organ is owing to previous predisposition in that organ, in consequence of which it suffers more than the rest *during* the general excitement which is raised in the hot fit of the ague, or the course of the continued fever.

The following are the conclusions to which M. Dufau has come, from the facts detailed, and many others which he has witnessed.

1mo. That there is such a thing as an intermittent irritation.

2ndo. That all, or almost all, the organs of the body are susceptible of this irritation.

3tio. That this irritation may be of a febrile or apyrectic nature—that is to say, it may or may not be accompanied by sympathetic symptoms.

4to. That intermittent fevers are probably febrile intermittent irritations.

5to. That the phenomena of these fevers may be produced by intermittent irritation of any or all of the organs of the body.

We acknowledge that the doctrine of our author is ingenious, and that it is much more tenable than that of either Broussais or Clutterbuck. We believe, too, that its adoption would not be very dangerous in practice; because, while it leads to gentle and antiphlogistic measures in continued fever—and particularly to local depletion when any organ is threatened, it excludes not tonics, and especially the bark, arsenic, &c. where the fever is of an intermittent type. It is decidedly a much safer theory than that of Brown, or even of Cullen; but we do not see that it possesses much, if any, superiority over the eclectic doctrine, which regards fever as a general disease, often shewing determinations to particular organs, in which determinations consists the principal danger—and to guard against which the practitioner should be ever on the watch in order to obviate the local inflammation by local or general depletion.

### 2. PHLEGMATIA DOLENS.

Dissection alone will not prove the pathology of a disease. It requires the evidence of symptoms during life, and also the light which treatment may be enabled to throw on the matter. In respect to phlegmatia dolens, it is certain that not one case in twenty proves fatal. If therefore, every twentieth case shewed, on dissection, inflammation of the inguinal vein, the cause of the original swelling of the limb would not thereby be proved to be phlebitis. In *fatal* cases, there may be this organic lesion found, and yet, in favourable cases, it may not have existed. One unequivocal case of phlegmatia dolens shewing the vessels of the groin *unaffected*, ruins the theory if supported by 20 cases in the affirmative. The following authentic instance has been put on record by Mr. Fraser, Surgeon of the Civil Hospital at Gibraltar, in the January Number of the Ed. Journal.

*Case.* A. M. 25 years of age, a stout woman, was admitted into the hospital on the 7th January, 1826, eight days after a confinement, having her left leg, from the toes to the groin, swollen to twice its natural size; and conveying to the touch—" an elastic, doughy, œdematous feel, but with no serous infiltration, as the parts do not pit on pressure." The tumefaction extended up along the same side, to the mamma and even to the axilla. The febrile symptoms ran high, and took on a typhoid character. We need not detail the treatment, which was judicious; but as the mamma sloughed, she was in considerable danger for some days, and then rallied and promised to do well. On the 15th January, however, there came on symptoms of thoracic inflammation, under which she sank on the 17th of the same month, ten days after admission into hospital. We shall pass over all parts of the dissection, except that which bears on the subject under discussion. All the abdominal viscera were sound except the uterus, which exhibited marks of inflammation, the vessels being large, and the organ itself the size of a pine-apple.

" On prosecuting the dissection, a chain of inflamed glands, from the size of a pea to that of a filbert, was seen extending from the distal point of the left iliac artery, amidst the cellular substance surrounding the sheath of the vessels, atlantad to the diaphragm, all as red as scarlet, and many purulent. The cellular substance around the iliac portion of artery also exhibited serous infiltration. The coats of the artery, with its vein, as well as the aorta, and *vena cava descendens*, were examined, but, upon slitting them up, not a trace of morbid action could be discovered. An incision was now made inside of the thigh down to the knee; the cellular substance was of the depth of two inches at the upper part, and infiltrated, as in the arm, but no serous exudation took place. Inflamed glands of the same description as related, skirted along the femoral sheath, from the groin to the popliteal space. In the groin three or four were seen as large as a pigeon's egg. The blood-vessels, as in the abdomen, were healthy. An incision was also made into the right, or sound thigh, and the contrast between the healthy and the diseased was remarkable. The lips of the vagina were much swollen." 18.

That this was a genuine case of phlegmatia dolens, there can be no reasonable doubt; and that it disproves the proximate cause which Dr. Davies has contended for, we think must be admitted. We have all along contended that, in phlegmatia dolens, there is no one structure peculiarly affected as the invariable origin of the disease. We think it very probable, however, that when the veins of the groin happen to become affected in the course of the disease, the danger is then much greater, and death more likely to result. In such cases, Dr. Davies' doctrine would appear to be supported, if not proved. But the numerous recoveries, without any particular treatment—and such cases as that brought forward by Mr. Fraser, disprove the exclusive pathology of inflammation of the veins being the primary cause of the disease.

The opinion of Dr. Hull, that the disease consists in a "peculiar inflammation seated in the muscles, cellular membrane, and inferior surface of the skin," appears to be as near the mark as any that has yet been offered.

---

### 3. EXAMINATION, POST MORTEM, OF TALMA.

Great contrariety of opinion existed among the medical attendants of Talma, respecting the nature of the disease which terminated this great actor's life. The partisans of Broussais maintained that he was affected with chronic "gastro-enterite"—while the others considered the enlargement of the abdomen, the nausea and vomiting, the great tenderness as well as tension of the parts, the obstinate constipation, &c. as more probably dependent on an organic obstruction to the free course of fæcal matters, and therefore predicted a fatal termination of the disease. Dissection proved the justice of this last opinion. There were present at the dissection (which was performed by M. Breschet) Messrs. Biett, Dupuytren, Fouquier, Broussais, and several others. Six inches from the termination of the rectum, there was found a stricture, and, in fact, a complete obliteration of the intestine. Above this stricture the rectum and colon were prodigiously distended, and in some places sphacelated and perforated, so as to give exit to stercoraceous matters into the cavity of the pelvis. The whole line of the intestinal canal was greatly distended with gas and fæcal matters. Traces of inflammation were found, of course, in various parts of the intestines, and were evidently the consequences of the retention of fæces. The stricture in the rectum was probably of long standing; for Talma had experienced, for years past, a difficulty in evacuating the bowels. He would frequently go in great haste to the water-closet, thinking there was a copious motion to come away, and yet, when the attempt was made, nothing but air, or a small quantity of liquid fæces would escape.

There was another morbid appearance on dissection, which is worthy of notice. At the apex of the heart there was a small aneurismal tumour or pouch, filled with concentric layers of fibrin, situated in the left ventricle, the parietes of which had grown remarkably thin at that part. It was about the size of a pigeon's egg. No symptom during life led to the suspicion of such a disease going on in the heart. But it was remembered that, one evening, after having enacted the part of Orestes in the play of Andromache, Talma felt himself strangely agitated, restless, and anxious for some time, which symptoms gradually subsided. But it is supposed that the internal membrane of the ventricle then gave way, and that the expedient of nature to fortify the part, by the deposition of coagulable lymph, was only a temporary or palliative cure.

About the time of Talma's death, the late General Kyd, of Albemarle-street, aged 73, died suddenly, without having evinced any symptom of disease of the heart during life. He was examined by Dr. Johnson and Mr. Hicks, who attended him for some years past, and precisely the same aneurismal pouch jutting from the left ventricle, as that described in Talma's case, was found. It was situated, however, near the base of the ventricle, at no great distance from the origin of the aorta. In the General's case, the centre of the aneurismal dilatation had given way, while the patient was asleep, and about eight ounces of blood were extravasated into the pericardium. The general had eaten a hearty dinner, and went to sleep, from which he never awoke. This excellent and universally respected officer had led a very active early life in India, whence he retired more than 20 years ago, to enjoy an age of ease, after a youth of labour. But soon after his return to Europe, he became hypochondriacal, and the malady increased as age advanced. For the last ten years of his life, he presented one of the most exquisite, though melancholy instances of this mysterious complaint that has perhaps ever been witnessed. Without exhibiting any mark of local or general disease, that was at all tangible, he became convinced that he was dying—or at least that he had but a few days or even hours to live. This too, at a time when he ate and drank heartily, and when all the secretions and excretions were healthy. He invariably, however, complained of an intense pain extending from the rectum, up along the line of the colon, and indeed along the small intestines, to the stomach. He never admitted that he was a moment free from this pain, though he would forget it sometimes for hours, when engaged in conversation with his friends. Whenever he recollected himself he reverted to the perennial source of his misery. It was discovered, some four or five years ago, that he had a small stone in the bladder, and it was now hoped that a key to this mysterious pain was found out. Sir Astley Cooper quickly removed the stone, but as soon as the wound was healed the malady returned as bad as ever. For a considerable period, before his death, the patient was almost invariably better and worse on alternate days. During the bad day, he would eat and drink very little, asserting that he was dying:—next day he would be better, and then he would eat and drink heartily—and so he went on. It is natural to suppose that the medical attendants were anxious to ascertain what was the state of the intestinal canal, in which the patient had complained of such intense pain for so many years. The body was examined with the greatest care, and not a vestige of disease could be detected in any portion of the alimentary passage, inside or out. The brain was also carefully dissected, but nothing unusual was observed. The aneurismal pouch of the left ventricle of the heart was the only deviation from apparently healthy structure in the whole body, and there can be little doubt that this was of comparatively recent date, and that it could have had nothing to do with the hypochondriacism of twenty years' duration, and with the feeling of agonizing pain in the intestines for more than ten years.

Here there was a neurosis of many years duration leaving no cognizable trace of disease after death. The sense of pain in the intestinal canal, however it might have been exaggerated by the patient, cannot be supposed to have had no existence. On the contrary, there is every reason to believe that the *unnatural sensibility* of the first passages was the cause of the hypochondriacism as well as of the pain in those parts. This supposition is strengthened by the fact, that the patient was always worse after free living, and always better after the day of abstinence.

General Kyd was a man of vigorous mind as well as body, and before he retired from active service, was of a remarkably cheerful disposition, fond of music, and every rational enjoyment, and yet temperate in the luxuries of the table. The sudden change to retirement soon began to shew itself in hypochondriacal feelings, and the patient endeavoured to amuse the mind by rambling over the classic scenes of Italy and Greece. But in this tour he did not pay so much attention to the physique as to the morale. He did not sufficiently exercise the body in proportion to his indulgence of the imagination in surveying antiquities, pictures, statues, &c. and the consequence was that he returned nearly as bad as when he commenced his tour. The due equilibrium between mental and corporeal exercise was not maintained, and little or no good resulted. The case altogether was a very instructive one for those who study the phenomena of hypochondriasis, and the multiplicity of features which it assumes in different individuals.

Before closing this short article, we may be permitted to express our surprise that the nature of Talma's disease was not more early discovered. A stricture within six inches of the anus was surely no very difficult matter to ascertain; and from the post-mortem examination there is every reason to believe that a timely use of the bougie would have prevented the obliteration of the canal, and consequently the death of the patient from that cause. Had the stricture of the rectum been discovered in time, and its closure prevented, it is highly probable that Talma, like Moliere and Palmer, would have expired on the *theatre* of his renown. This would have been a much easier death than that to which he was doomed by the terrible malady in question, and much more in consonance with the life of this great tragedian.

### 4. VIPER-BITE.*

*Case.* Tourfant, an herbalist, aged 45 years, who had been occasionally attacked with epileptic fits, at long intervals, was bitten by a viper, on the 17th of May, 1826, at half-past 5 in the morning. The reptile was presented to him for sale by a peasant of Fontainbleau, and the wound was inflicted between two of the metacarpal bones of the right hand. There was another smaller wound near the former, but of uncertain nature. The animal bit another man a short time afterwards; but on applying some oil and sal-ammoniac no bad consequences ensued. At 7 o'clock, Tourfant's hand swelled and became painful, and at 8 M. Piorry and M. Solon saw the patient. The wound was extremely small—the integuments of the hand enormously swelled, but not very painful—skin of the member pale, with a slightly violet tint, menacing immediate gangrene. The temperature of the parts was considerably below par. The man vomited, from time to time, some mucous and bilious matters—and passed by stool some motions of a peculiarly fetid odour—pulsations of the heart extremely feeble—those of the carotids could not be felt, nor those of the radial. The crural arteries, on the contrary, pulsated strongly. The upper extremities and the head were of an icy coldness, whilst the abdomen and lower extremities were preternaturally warm. The face was nearly as much swollen as the hands—the lower lip livid, and the neck in a similar state with the face. The patient still preserved his intellects, but was dreadfully depressed in spirits, being scarcely able to speak. There was no spasmodic or convulsive movement in any part of the body.

An incision, three lines in length, was made into each wound, and a cupping-glass, with a pump, applied. No blood, but only a serous fluid, was drawn out. With this fluid, a young cat was inoculated, but no bad consequences resulted. The cupping-glass was kept applied for more than half an hour, during which, some spoonfuls of serosity were discharged. Warm fomentations were applied to the lower extremities, and the arms and region of the heart were chafed with warm frictions. Diluent drinks only were exhibited. In half an hour the patient spoke stronger—the infiltration of the face had subsided a little. At nine o'clock, the medical attendants again returned to the patient, and found the symptoms somewhat ameliorated. The pulsations of the radial and carotid arteries were still imperceptible. The cupping-glass was re-applied, and a considerable quantity of serous fluid extracted. In the course of the day, the tumefaction of the face and upper extremities abated much—the action of the heart became more developed—the nausea disappeared—and the pulsation of the carotids and radials again became tangible. In the evening, the face was void of swelling—the glands in the axilla were swelled and painful—and an erysipelatous blush extended along the arm in the course of the lymphatic vessels. The action of the heart and arteries was now strongly developed. 18th. All the constitutional symptoms had disappeared: but still the arm was

* M. Piorry. Revue Med. Octobre, 1826.

double its natural size, very red, and very painful on pressure. The whole member now appeared to be the seat of a vast phlegmonous erysipelas. Twenty leeches were applied, followed by poultices. The diet was rigorous. In the evening the symptoms had much abated. From this time every thing went on well till the evening of the 20th, when much mental agitation occurred, and next day the patient was delirious, without fever. Something of this kind had formerly been observed for some days after each attack of epilepsy; but no fit had occurred this time. 22d. The cerebral commotion became subdued, and the patient continued to do well. Several medical practitioners had seen the patient during the treatment.

The above case appears to our author to justify the conclusion, that the cupping-glass is of essential service in poisoned wounds; and it is, we believe, the first instance where this remedy was solely trusted to in the human subject, at least in modern times. Our author pays a well-merited tribute of applause to the exertions and genius of Dr. Barry, in recalling this ancient practice, which had slept in oblivion so many centuries. We were not aware that the cupping-glass had been used so late as the present century. It appears, from M. Piorry, that in a dissertation, by M. Blot, (date not mentioned) two cases of viper-bites were treated by M. Guyon by scarifications, cupping-glasses, and cauterization. Both were cured. In the New Journal of Medicine, for 1820, we find a case recorded by M. Richard, where a cupping-glass was applied over a poisoned wound, but did not arrest the progress of the virus. The particulars of the application, however, are not satisfactorily detailed.

M. Piorry enters into a long discussion on the mode of action of the viper poison; and whether it is to be considered as exerting its influence through the medium of the nerves or the blood. He comes to the general conclusion, now almost exclusively adopted, that the poison is absorbed, and does not act through the medium of the nerves. M. Piorry next takes up the subject of atmospheric pressure as the *sole cause* of the venous circulation—"La cause *exclusive* de la circulation veineuse"—and decides in the negative. It is astonishing what a propensity there is in human nature to misinterpret the plainest propositions. Indeed, we might almost lay it down as an axiom, especially in medicine, that no man will be rightly understood, even if he were to say that two and two make four. On both sides of the Channel, Dr. Barry is understood, by nineteen in twenty, as maintaining that atmospheric pressure is the sole cause of the motion of the blood in the veins. Yet that gentleman not only does not maintain any such thing, but does not even place the agency in question as the principal of many causes which tend to the same end and object. In his work he distinctly makes the *vis à tergo* occupy the *first* rank, and places the suction influence (or, more correctly speaking, atmospheric pressure) in the *second* rank. M. Piorry, therefore, like many of his brethren, has set up a man of straw, which he very triumphantly knocks down, and thinks himself very clever in so doing.

As M. Piorry, however, allows that atmospheric pressure is *one of the agents* in the venous circulation, Dr. Barry's theory stands exactly where it was. M. Piorry grants that this agency is secondary—" cette influence est secondaire." Dr. Barry does the same. The plus or minus of this agency will never, perhaps, be precisely ascertained. It is sufficient that it is acknowledged to exist.

---

### 5. BISCHE—A MALIGNANT DYSENTERY OF TRINIDAD.*

The first case which Dr. O'Connor met with, of this strange and dire disease, was in the person of a young Indian (Mœlissa) girl, ten years of age. She had then been three days ill. During the first two days, she had suffered from violent and constant irritation of the stomach, and dysentery. She was under medical treatment, when, " fortunately," an old experienced nurse was called in, and quickly recognized the nature of the disease, and prescribed the specific remedy—strong lemonade for drink, and suppositories of limes, soot, and some other ingredients of no material efficacy. This treatment had been commenced four hours before our author saw the patient, and the symptoms were said to be much mitigated. Her then condition was as follows:—pulse scarcely perceptible—skin cold and clammy—vital powers apparently fast sinking—sphincter ani so relaxed, that a suppository, the size of an egg, was easily introduced. Our author had not the slightest hope of recovery; but not so the nurse. He, therefore, patiently waited the result of the vegetable-acid treatment. To his astonishment the female recovered, though the convalescence was tedious. Our author then made enquiries respecting the nature of the disease; but could gain little other information than that it was a flux, which invariably proved fatal, unless abundance of lemon juice was used, both by the mouth and per anum.

Dr. O'Connor had no good opportunity of seeing the disease again till the year 1823. On the 24th July he was summoned to the relief of a negro, a robust man of good constitution, about 26 years of age. He had been two days ill, and entered the hospital of Belle Plaine Establishment that morning. Our author had seen him on the 22d, being then in good health, and was quite astonished to perceive the immense emaciation that had taken place in two days. The man was greatly depressed in spirits, and seemed confident that the disease would terminate fatally, as he had neglected the Bische, and nothing now could save him. His pulse was 136, and scarcely perceptible, skin cold, and covered with clammy perspiration, countenance dejected, alvine and urinary excretions passing away involuntarily and frequently, the former mixed with blood and mucus. On examination, the sphincter ani was found completely relaxed, and he could compare the anus to nothing

---

* Medical Observations on a Disease known in the Island of Trinidad, by the name of Bische, or Biecho. By I. L. O'Connor, M.D. Assistant-surgeon, Royal Artillery. [Analyzed from a MS. account forwarded to the Editor.]

else than the sunken eye of a dead animal in an advanced degree of putrefaction. The suppository of lime-juice had been freely used during the preceding twelve hours, without any effect, and the disease was rapidly advancing, the poor man suffering excruciating pain. Our author ordered him half a grain of opium and seven of calomel every four hours, with injections of arrow-root and laudanum, after which, lime-juice suppositories were to be introduced. Brandy-punch for common drink. 25th. Passed a restless night—suffers excruciating pain in the rectum. Has taken 35 grains of calomel and three of opium, besides half an ounce of laudanum in glyster. He refused to take any more medicine, and requested to have a Spanish nurse, in which he was indulged. She gave him lime juice and some other unimportant things. 26th. The anus has sloughed, and the sphincter is partly destroyed. The nurse had passed more than a pint of lime-juice up the rectum, with as much facility as into a funnel. 27th. Is sinking rapidly, and prays for death. From the scrotum to the os coccygis there is only one foul sore, like the face of an ill-conditioned stump after amputation! One could see several inches up into the cavity of the abdomen. Opium gave no relief, and the wretched sufferer was always exclaiming—"I "am eaten up by the Bische; and he is so hungry, that he never stops, "day or night; and when he stops, he and I die together." He died that night, and the body was examined 26 hours after death. The putrefaction, however, had made such advances that nothing satisfactory could be gleaned from the dissection. There were no worms in the intestines.

The disease is attributed by the natives to an insect which lays its eggs in the rugæ of the rectum when the person evacuates his fæces, and protrudes any part of the mucous membrane, in a woody or jungly spot. Our author has never seen any proof of the existence of this insect, and is more inclined to consider the malady as a species of dysentery commencing in the rectum, and confined to the lower portion of intestine. When taken early, its progress is easily checked, in the same way as gonorrhœa or ophthalmia; but if the disease be once communicated to the cellular structure surrounding the gut, the destructive progress goes on to a fatal termination. It appears principally among the African and Indian races. Our author met with one instance, however, in the person of an English half-pay officer. It was quickly subdued by the application of lime juice.

In our author's practice in Trinidad, he made a free use of lime juice, whenever the evacuations were bloody and mucous, and with decided benefit. This practice is adopted by the most experienced practitioners of the colony above-mentioned.

Dr. Ferguson, of Windsor, through whom we received the above curious document, has appended many ingenious and practical remarks, which our limits prevent us from giving at length. He looks upon the disease as a form of malignant dysentery, and asserts that he has seen many instances where the rectum was similarly affected among our soldiers in Flanders, during the campaigns of 1794, and subsequently in various parts of the world. Dr. F. speaks highly of the utility of lime-juice and ripe fruits in dysentery, and mentions that the late Dr. Cabell was so much struck with the efficacy of this class of remedies in Spain, that when severely attacked with dysentery afterwards in Portugal, he rejected all medicine, and trusted his own cure to the abundant use of lemons and oranges. He recovered, and afterwards pursued the same treatment in the hospitals of the British army. We shall conclude this article with the following extract from Dr. Ferguson's comments on the paper of Dr. O'Connor.

"One word respecting the general treatment, and I have done:—which is, that, of all the plans offered for the cure of dysentery, the mercurial is unquestionably the best; not only because through its alterative accumulative powers, it supersedes almost every inflammation that is not too rapid in its course to be overtaken by any but the speediest remedies; (witness the hepatic, the syphilitic, the rheumatic, the iritic, and other inflammations;) but because all the common forms of mercury can be made to coalesce most advantageously with the anodyne, the sudorific, and the depletory auxiliaries; or even with the citric acid through the aid of opium, and still better if the skin be chosen for the medium of its introduction."

---

### 6. FATAL ABDOMINAL INJURY.

A melancholy case of this kind is reported from Guy's Hospital in No. 174 of the Lancet, accompanied by much implied censure on the practice of the medical officers in attendance. We may here remark, that hospital, like police reporters, should be cautious how they exhibit *themselves* on the stage. It has been well observed by the Morning Chronicle, that "*the business of a reporter is not to furnish a farce for the public, but to give an honest account of proceedings.*" Again, "*the reporter should be passive, and not appear in his own character as a satirist.*"* This rule is still more necessary in hospital reports, where the character of medical officers may be injured by a licence of the pen of the reporter, indulged in perhaps from caprice, or founded on error or misapprehension. We do not say that the report which we are about to notice is incorrect; but we think the bare statement itself would have answered all good purposes, without any italics, notes of admiration, or censorious comments of the reporter.

The case was that of a healthy young countryman, 23 years of age, who, half an hour previous to admission, fell from the top of a loaded waggon to the ground, a distance of 20 feet. It did not appear that he struck against any thing before he fell on his back, on the ground. When admitted, (Nov. 25, 1826,) the man was pallid, and "the *surface* of the body and the pulse at the wrist very *feeble*." In an hour or two, there was some return of warmth, and he vomited a dark brown

---

* Morning Chronicle, Jan. 5, 1827.

fluid, complaining of great pain in the abdomen, especially in the right iliac region. The abdomen was tense and tender, the muscles appearing to be rigidly in action—pulse small and jerking at the end of three hours. He was ordered to be cupped on the loins—to have a purgative enema, and to take a grain of calomel with half a grain of opium, every five or six hours. 26th at noon; the vomiting continued through the night, but ceased this morning—pulse 120, and oppressed—tension and tenderness of abdomen continue. Sixteen ounces of blood were ordered from the arm, after which, the pulse became more expanded for about two hours, when it resumed its former character. 4 p. m. Fomentations to the abdomen—purgative enema. 27th. The pain and tenderness of abdomen undiminished—little sleep in the night—pulse 130, and small—tongue dry and brown—occasional vomiting. Opium given. 28th. Pulse 120—vomiting has ceased—bowels opened. One grain of opium. 29th. Pulse 130—abdominal symptoms still the same—bowels much purged. The cretaceous mixture, with confect. aromat.—twelve leeches to the abdomen—opium in the evening. 30th. Diarrhœa excessive—pulse 125—countenance flushed. Laudanum by the mouth and per anum. Dec. 1. Pulse incompressible and very sharp. To be cupped on the loins to six ounces—laudanum given for the diarrhœa, which is still excessive. Dec. 2. Things much the same; ten leeches to the iliac region. The patient lingered till the 7th, when death put a period to his sufferings.

*Dissection.* On opening the abdomen, on the right side, "air escaped, with a large quantity of yellowish fluid, partly *fæcal* and partly purulent." This came from an extensive abscess formed "in front of the peritoneum." The "boundaries of this cavity were formed by preternatural adhesions. It was well defined, and was very extensive, reaching above as high as the liver, and below to the right iliac fossa." Another extensive abscess was found behind the peritoneum, extending over the lumbar muscles, as high as the eighth rib. The peritoneal covering of the intestines was generally inflamed. "There was *no lesion* of the intestines at any part *to be* detected; and, if any had taken place *in the peritoneum,* on the right side, in consequence of the fall, it could not be discovered in its diseased state."

The reporter's account of the *post-mortem* appearances is any thing but lucid. How he could first state that fæcal matters rushed out on opening the abdomen, and afterwards entertain a doubt of perforation of the intestine, we are at a loss to imagine. When we consider the height from which the patient fell—the symptoms of alarming collapse which he quickly evinced, the pallid countenance, cold skin, feeble pulse, vomiting, and tension of the abdominal muscles—and couple all these with the appearances on dissection, we cannot for a moment hesitate to believe that the intestine was ruptured by the fall, and that no treatment, however active, would have saved the unfortunate man's life. That such men as Mr. Callaway and Mr. Key adapted their plan of depletion to the phenomena which the patient presented, we have no doubt; and therefore, viewing the whole case, we absolve them freely from that censure which is meant to be conveyed by their harsh—we had almost said *unjust* judge, the reporter, in the following concluding line of the case:—" *Passive* treatment for *active* inflammation."

As it is our intention to take a critical survey of all the more important hospital reports in the LANCET, we solicit information, from the proper sources, on all points in the said reports, where error, misconception, or misrepresentation may have mingled themselves with the narratives. By this measure, we hope to contribute equally to public justice and public utility.

---

### 7. POST-MORTEM CHANGES IN MUCOUS MEMBRANES.

In our last number (11) pages 295-7, we presented our readers with a suite of experiments, made at the Veterinary School of Alfort, by Messrs. Rigot and Trousseau, respecting the *post-mortem* changes which take place in the internal surfaces of the blood-vessels. We now proceed to the experiments which relate to the internal surfaces of certain important canals in the animal fabric.

Most authors who have written on the pathology of the alimentary canal, have thought it proper to describe the appearances in a state of health. For this purpose the bodies of criminals have been examined, and, strange to say, an exceeding discrepancy of opinion was soon found to obtain in regard to the natural colour and appearance of the intestinal tissues! One author represents the mucous membrane of the stomach and bowels as of a yellowish white colour, mixed with a rosy tint:—Another says, the said membrane presents a vivid red colour; while a third affirms, that the natural colour of this tissue is grey, &c. When such differences were found in the parts in question, it was natural to inquire whether there were not some causes to be assigned for these varieties of appearance. In France, criminals are beheaded, and there is great hæmorrhage:—The mucous membranes are there generally found pale. In other countries, as in England, where criminals are hanged, the said tissues are often found of a vivid red tint.* Again:—Considerable difference was found in the colour of different parts of the same intestine—the superincumbent and the subjacent portions, for example. The *former* are often found pale, and the *latter* red. Each has been asserted to be the natural or healthy colour during life—but without sufficient reason.

Among those who have specially directed their attention to this subject, M. Bretonneau of Tours, must be particularly mentioned. He not

---

* In our last number, page 49, while reviewing Dr. Paris's work, we gave an example of the want of attention to the circumstances attending death, while reporting on diseased appearances on dissection. Thus, even Dr. Paris wonders at the paleness of a man's brain, where death was occasioned by hæmorrhage, and speculates on this paleness as evincing proofs of a *deficient circulation* in the brain during life! If physicians of Dr. Paris's character will commit such oversights in pathological investigations, what may be expected from the general run of investigators?

only examined the intestinal canal in those who had been executed, and who had died suddenly, while in apparent health ; but also in those who died of diseases situated in other parts of the body. This was the true path to pursue. A man, for example, who was in previous health, is seized with pneumonia, and dies in seven or eight days, presenting no other symptoms than those characteristic of pulmonic inflammation. If, on dissection, the redness of tissue (which we shall presently describe) appear in the gastro-intestinal mucous membrane, we cannot, without perplexity, consider it as a proof of previous inflammation; for if we do, we must admit the existence of diseases without their symptoms, and thus be led into a Cretan labyrinth. Those who infer inflammation from the appearance of redness, and without symptoms, beg the question, and fall into the most inconceivable errors. Prost having discovered this redness in the lining membrane of the bowels, in almost the whole of those who died in the Parisian hospitals, declared it to be the effect of inflammation : Broussais raised this into a doctrine which exercises a wide and almost unbounded influence on the minds of men. He has thus given to his "*gastro-enterite*" too important a station in the nosological chart.

The authors of the present suite of experiments have endeavoured to throw light upon this important subject, by careful examination of healthy animals *immediately* after death, and at certain periods after the same event. A number of horses and dogs were killed, for the sole purpose of ascertaining what is the natural and healthy appearance of the mucous membrane ; and immense differences were observable between the said tissues in dogs and in horses. In the former class, whose intestines are furnished with long and numerous villi, the mucous membrane was generally found of a tint resembling the lees of wine, this tint being more or less vivid, according as the membrane was in contact with alimentary matters, or only simply with mucous secretions. The villi themselves were usually pale, and the colour seemed seated in either the chorion, or the subjacent cellular structure. The tint abovementioned varies much, according to the time which elapses during the examination. A few minutes made all the difference between paleness and redness, in knuckles of intestine placed under exactly the same circumstances in all other respects. This difference was no doubt owing to the action of the air—not, our authors think, the *chemical* action on the blood in the vessels—but the effect of an increased peristaltic movement, under the influence of the air, by which more blood is poured through the vessels of the surface thus exposed.

The experimenters are of opinion that the intestines of dogs are not calculated to afford just ideas of the natural colour of these parts in man. On some rare occasions, where abdominal wounds have given issue to the human intestines, the coats have appeared much thinner—the villi less discernible—and the blood-vessels much more numerous in the human than in the canine subject. The intestines of the horse, however, bear a considerable resemblance to those of man, both as to the thickness of the coats, the size of the villi, and the distribution of the blood-vessels.

The resemblance, indeed, between the equine and human intestines is not perfect. If the mucous membrane of the dog is more coloured than that of the human subject, that of the horse is rather less so. They examined 25 horses, which might be said to be alive, as the heart continued to pulsate till half the track of intestinal canal was overhauled. On all portions of intestine, examined while the heart was yet beating, they could plainly see the meseraic vessels externally playing over the gut; but not in great numbers. The peritoneal covering never exhibited the smallest colour or trace of injection. The parietes of the intestines appeared much thicker than in the dead animal, and the calibre much less in size. The colour of the mucous membrane was generally pale, with a slightly yellow tint. In some places there was occasionally seen a very faint reddish tint. The internal surface of the stomach had rather more of this tint than the intestines.

We cannot afford space for the details of the individual experiments; but we perceive that the redness of the mucous membranes increased in proportion to the time which elapsed between the death of the animal and the dissection—also, according to the position of the parts, the superimposed being less, and the subjacent portions more tinged. The redness appeared evidently to be occasioned by the injection of the capillaries from the contraction of the arterial trunks, which contraction has been shewn by Dr. Parry to go on for some time after cessation of the heart's action—in fact, till the arterial tubes have nearly or completely emptied themselves. Gravitation, also, has great influence in producing congestions of blood in all depending parts.

Our authors are indefatigably employed in ascertaining the differences between *post-mortem* and *ante-mortem* changes in the tissues of the body, which they will publish forthwith. In the mean time, they consider themselves authorized from the numerous experiments which they have already detailed, to draw the following conclusions :—

1mo. That the arborescent injection, occupying the trunks, branches, and ramifications of the meseraic veins, is the invariable result of a stasis of the blood in the subjacent or dependent knuckles (dans les anses déclives) of the intestinal tube.

2ndo. That, generally speaking, this injection does not go beyond the ramifications of the veins, (ramuscules veineux) in the bodies of healthy individuals.

3tio. That often, in those who have died of an acute disease, (provided they were not profusely bled) the trunks, branches, and ramifications of the veins are injected, as also the villi, without any inflammation having existed, during life, in the intestine.

4to. That sometimes the blood distends, at once, the trunks, branches, and ramifications of the veins—colours the villi, and transudes on the surface of the mucous membrane, without any inflammation of the intestines, merely from stasis of the blood in depending parts.

What has been said of the small intestines will apply also to the stomach and to the colon, excepting that, in these latter, the appearances are not so well marked. These appearances have also *proved*, what, indeed, has been long suspected, namely, that the *post-mortem* or cadaveric alterations in the lungs may go to such an extent as to diminish the cohesion of the pulmonary parenchyma, and completely expel the air from the air-cells of dependent portions of lung. They prove, also, that the bronchia may become vividly coloured in the parts of lung where the blood has accumulated, without any preceding inflammation.

We recommend these experiments and observations to the attentive consideration of the profession; and hope they will tend to correct many erroneous conclusions which are daily drawn by heedless inspectors of the dead.

---

### 8. DISEASE OF THE THYMUS GLAND.

In the 5th No. of the Ed. Journal of Medical Science, Mr. Wood, of Kilmarnock, has published a paper, entitled "Cases of Sudden Death, and Affections of the Head, originating from Disease in the Thymus Gland and Chest." This paper is illustrated by the details of nine cases, with the dissections, and a long train of observations. We shall first give the conclusions which Mr. Wood has come to, from these data, and then introduce the particulars of one or two cases.

"From the facts which have been presented in the history of these cases, and the appearances which were exhibited on dissection, the following inferences may be drawn:—

"*First*, That the thymus gland is subject to disease, and susceptible of undergoing various morbid changes, such as scrofulous enlargement, scrofulous ulceration, and caseous degeneration.

"*Secondly*, That diseases of the chest, sometimes in adults, and enlargements of this gland in children, frequently induce a dropsical condition of the brain, without being preceded or accompanied by the idiopathic symptoms of hydrocephalus.

"*Thirdly*, That morbid enlargement of this gland may be inferred to have existed during life, when the child is cut off suddenly, without any previous complaint, from a fit of crying, or violent irritation of any kind, however it may have been induced.

"*Fourthly*, That chronick enlargement of this gland may be inferred to exist in infants subject to fits, as in the first, third, and seventh cases, and in all cases of chronick, spasmodick, or cerebral croup, as this complaint has been variously designated by different writers.

"*Fifthly*, That in all these chronick cases, water in general will be found in the ventricles, and on the surface of the brain, in consequence of the pressure of the gland upon the trunks of the veins coming from the head.

"*Sixthly*, That a dropsical condition of the brain, proceeding from venous exudation, may be cured, if the disease in which it originated can be removed, and in the attempt to effect this, the patient must be treated in every respect as for an enlarged scrofulous gland."—*Journal*, p. 69.

We shall particularize a few cases, in order that our readers may be enabled to judge how far Mr. Wood is justified in the conclusions (somewhat extraordinary certainly) which he has drawn.

*Case* 1. A child, aged 9 months, "was seized with a sort of convulsive disorder," in November, 1814, which was removed by the warm bath, leeches, and some common remedies. The paroxysms returned occasionally, especially when the child was awoke suddenly out of a sleep—or when it cried vehemently. It then became livid, and was incapable of making a full inspiration, its instinctive efforts to draw in breath being stopped and broken by convulsive struggles, reiterated at short intervals. The evening before its death, the child was lively, and had the aspect of good health. The following morning, as the child was lying supine, crying on its mother's knee, it suddenly expired in a convulsion.

*Dissection.* The blood-vessels of the meninges were much distended, though considerable effusion had taken place from the veins. Both hemispheres of the brain were dropsical on the surface, a quantity of serous fluid having been effused between the pia mater and tunica arachnoidea. The brain itself was soft, and in the right ventricle there was half an ounce of water. In the chest, the heart, lungs, and large vessels were sound; "but the thymus gland was much enlarged, and, as it was supposed, might weigh considerably more than two ounces." On cutting into its substance, a quantity of thick brownish matter escaped, resembling ill-formed pus.

*Case* 2. This was a child, only 13 months old, who died suddenly in consequence of an incident apparently trifling. He was supposed to be in good health, and of sound constitution. When the child cried vehemently, or was suddenly started from sleep, its countenance became livid, and its respiration impeded.

On dissection, the lungs were sound, but the large veins in the top of the chest were extremely distended with blood. The thymus gland might weigh two or three ounces, and was partially indurated. It contained a creamy purulent looking matter. The head not examined.

Seven of the nine cases occurred in children—two in adults. We shall give a short analysis of the latter.

*Case* 3. (*Case* viii.) A woman, aged 58 years, complained, for more than a year, of pain immediately behind the sternum, not so severe as to prevent her from attending to her domestic concerns. A cough imperceptibly arose, and for the last three months of her life had a loud, croupy sound, attended with difficult respiration. Mr. W. suspected the pressure of some tumour on the trachea. Leechings and an alterative mercurial course were prescribed. While the leeches were sucking, some relief was afforded; but no sooner had they dropped off, than all the symptoms returned with tenfold violence. The veins of the head were seen greatly distended, and the obstruction to respiration was great.

In this state she continued about eight days, without swallowing any nourishment, and suddenly expired at last.

*Dissection.* The lungs were sound—heart soft and fatty—arch of the aorta aneurismal, the tumour pressing on the bifurcation of the wind-pipe. There was much infarction of the cellular substance between the tumour and the descending cava, apparently obstructing the free return of the blood from the head. The brain was not examined.

*Case* 4. (ix.) A man aged 60 years, who had enjoyed good health, was seized with a fit of laughing, in a work-shop; and when the cause of mirth was over, still he continued involuntarily to laugh. He remained in this state till he fell down in a fit of epilepsy. For several weeks he had returns of the fits, to which succeeded a kind of mania. This last continued six or eight weeks, and then gradually subsided, when he was seized with asthma so severe as to be little susceptible of alleviation. He gradually sunk under this disease.

*On dissection*, the vessels of the head were found distended, and there was effusion of water on the surface of the brain. The ventricles contained a large quantity of water. On raising the sternum, a white hard tumour of great magnitude was observed lying to the right of the base of the heart. The veins on the top of the chest were much distended with blood, and the tumour was found to embrace the whole trunk of the descending cava, thus greatly diminishing its calibre. A white cauliflower looking substance also projected into the inside of the vessel an inch above where it enters the auricle. The tumour weighed nearly two pounds, and involved the trunk of the pulmonary veins. It was of firm consistence, but when cut into, a cream-coloured fluid issued, not contained in any particular sac. There was no other disease.

Our author enters next upon a review of the nine cases which he has detailed, and considers that one general feature pervades the whole, modified by the particular circumstances of each individual. "In all the seven children, the thymus gland seemed to be the primary seat of the disease. In four of these it was very much enlarged; in one case it was caseous, in another, ulcerated, and in a third, it appeared to have been entirely destroyed by a process of ulceration." In the aneurismal patient, "the croupy cough, the insupportable dyspnœa, the swelling of the veins in the neck and arms, evince with how much difficulty the blood was transmitted through the circulating system." The large tumour in the last case, by obstructing the return of blood from the head, produced, in our author's opinion, asthma and effusion of water on the surface of the brain. "The effusion of water on the surface of the brain and in the ventricles, in the majority of the cases, in which this viscus was examined, appeared evidently to be the *consequence* of some obstruction to the return of the blood by the veins from the head, rather than the effect of inflammation in the brain itself, or its membranes."

It is upon this last pathological supposition, that the whole drift of Mr. Wood's paper rests. We confess that the facts and arguments brought forward by Mr. Wood, have not carried conviction on this point. Contemplating the serious morbid appearances in the brains of these children, we are led to the inevitable conclusion, that their sudden deaths were owing to the cerebral affection; but that this cerebral affection was owing to previous disease in the thymus gland, we have very great doubts. Nevertheless we give all due credit and praise to Mr. Wood for the candid manner in which he has detailed the facts, and for the ingenuity with which he has maintained his own theory respecting the etiology and pathology of the fatal diseases here brought before the notice of his brethren. We recommend an attentive perusal of the original to those who are desirous of further information on the subject.

---

### 9. FATAL MORTIFICATION OF THE GREAT TOE.

A curious and melancholy case of this kind was lately reported [Lancet, No. 167] from Bartholomew's Hospital. The patient had been a sailor, and received a bruise from a horse's foot, on the toe, some three weeks previously. The toe had swelled, became livid, and festered. The constitution began to sympathize in a week after the accident. A fortnight before he was admitted, the toe became insensible, after having been very painful. *Nov.* 2. The integuments of the toe are livid or black—and beyond this, the skin is of a dusky ash-colour, and vesicated—no distinct line of demarcation. Constitution greatly disturbed—head-ache distressing—no sleep—pain in the foot unremitting. He was ordered aperient medicine, and after its operation, wine and quinine. 3*d.* He passed a better night—was this morning free from head-ache, and had a clean tongue. Towards night the bad symptoms returned. The wine and quinine discontinued. 4*th.* The above medicines were resumed, and continued for some days. At one time there was a faint appearance of commencing separation; but this was transitory, and the gangrene spread slowly upwards. On the 14*th*, although the mortification was still spreading, yet the foot and leg were much less swelled—the tongue was clean—and the pulse was down to 90, with good appetite. Mr. Lawrence called Messrs. Earle and Vincent to consult respecting the propriety of amputation. Mr. L. and Mr. Earle were for the operation—Mr. Vincent against it, considering there was ossification of the arteries, and observing that, in three similar cases, amputation was unsuccessful. On the 18*th*, things were nearly in *statu quo*, but the mortification was still spreading. Mr. Lawrence determined to amputate, and the operation was performed below the knee. There was much oozing of blood from the small vessels, and *two veins* were tied. The arteries were found somewhat diseased. Hæmorrhage came on, in the evening of the operation, and was restrained by cold. On the 20th the stump was opened, and no adhesion had taken place. There was an offensive sanious discharge. He lingered till the 3d December, when he died.

On *dissection*, the arteries were found, here and there, ossified—vena saphena interna thickened and resembling an artery. In this and in the femoral vein there was purulent matter, but no appearance of inflammation. In both hip-joints there was pus in the cavity of the capsule, and the reflected portion of synovial membrane was absorbed, and the cartilage ulcerated. In the thorax there was some sero-purulent effusion.

In this case Mr. Lawrence and Mr. Earle acted on the principle recommended many years ago by Baron Larrey—that of not waiting for a line of demarcation between the dead and living parts. In two instances we adopted this practice, when the mortification was threatening to extend to the upper part of the thigh; but in both, we failed of success. We believe, with Mr. Vincent, that the practice is inadmissible. If the constitution has not power to arrest the progress of the mortification, we do not think that the addition of an operation will augment that power, or prevent the morbid process going forward afterwards in the stump. Baron Larrey, however thinks differently, and has adduced cases of success.

### 10. OBLITERATION OF THE AORTA.

Dr. A. Monro has communicated to the Editor of the EDINBURGH JOURNAL of MEDICAL SCIENCE, some particulars of a case where the abdominal aorta was obstructed by a tumour. The man was of middle age, and having been exposed to cold, was seized with pain of side, difficulty of breathing, cough, expectoration, and other symptoms of phthisis, of which he died in the course of four months. He had evinced no degree of weakness, numbness, or palsy of the limbs, and could walk till within a day or two of his death. When the abdomen came to be examined, a circumscribed tumour was observed lying upon the second and third lumbar vertebræ, connected with the aorta. It was about the size of an orange flattened, and adherent to the second and third vertebræ, which were carious. It was filled with clotted blood. The sac did not seem to be composed of distinct layers, but resembled thick leather that had been soaked in water. The aorta entered into the middle of the sac, and the latter appeared to be a uniform expansion of the former, at its division into the iliacs. The tumour was filled by layers of coagulable lymph crossing each other in various directions, so as to form a confused and irregular mass. The contracted portion of aorta immediately below the aneurism was quite impervious. The case therefore proves that the circulation may be carried on below an obliteration even of the aorta, by means of various anastomosing vessels. The case goes to the justification of the attempt made by Sir Astley Cooper to save life by tying the aorta. The want of success in one or two cases is no proof that the principle is bad. At the same time, we can hardly expect that the system will bear a formidable surgical operation, and sudden ligature of the aorta, with the same degree of impunity as a gradual obliteration of the vessel by an aneurismal tumour. These spontaneous obstructions and the results of experiments on animals merely form, as we said before, justificatory data for the aortic ligature, in cases of extreme danger, and where no other means can offer a chance of saving life.

### MEDICAL JURISPRUDENCE.

### II. HOMICIDE—SUICIDE—INFANTICIDE.*

The crimes at the head of this article are every day attributed to insanity—and especially to a peculiar monomania, or irresistible propensity in individuals to imbrue their hands in the blood of themselves or others. That homicide is *sometimes* committed through this propensity, there can be no doubt—that infanticide is *often* perpetrated during a momentary subversion of reason and conscience, we believe—and that suicide is *almost always* the result of intellectual aberration, on one or more particular topics, we have very little doubt.

The actions of men are obvious enough; but the motives are generally concealed in impenetrable night. As far as regards homicide and infanticide, the strictest scrutiny should, of course, be put in force, when the plea of insanity is urged; but we have ever entertained a horror of that law which pursues the suicide beyond the grave, and stabs the lifeless corpse, not indeed as a punishment for the crime committed, but as a preventive of the crime in others. That the infliction of the penalty on the body of the suicide would have the least effect in deterring others, we cannot admit, since it has been proved that even the accounts which are published of suicide and infanticide have frequently led to the same catastrophes in others, by the impressions made on weak minds, thus leading to subversion of reason. The plea of insanity for the extenuation of crimes subjects the medical witness to a most embarrassing examination, and places him in a situation of great difficulty. The most effectual mode of preparing himself for such scrutinies is to study individual cases first, and draw general conclusions afterwards. It is on this account we have taken up the recent publication of M. Georget, who has published some "*causes celebres,*" where this mono-maniacal propensity was pleaded, and where great general and professional interest was excited in France by the said trials. Contrary to the plan of M. Georget, we shall place an analysis of these trials before our readers before we enter into any discussion of the subject generally.

### TRIAL OF HARRIET CORNIER.

The case of this female produced an uncommon sensation at the time, and it has been publicly asserted, on the best authority, that the reports of her trial led to the commission of several similar crimes.†

---

* Discussion Medico-Legale sur la Folie, ou Alienation Mentale, suivie de l'Examen du Procès Criminel d'Henriette Corniere et de plusieurs autres Procés dans lesquelles cette Maladie a eté allegué comme Moyen de Defense. Par M. Georget, M.D.

† See pages 226-7 of our last Number, where some of these cases are detailed.

H. Cornier a female servant, aged 27 years, had been of a cheerful and rather gay disposition, till the month of June, 1825, when she was perceived to become taciturn and melancholy. Not giving satisfaction to her employers, she was discharged their service, and from that period her melancholy increased to a kind of stupor. She would not disclose to her relations the cause of her mental dejection. One morning in September (1825) she came to the house of a female cousin, pale and agitated, stating that she had just made an attempt to drown herself in the Seine, but was prevented by some persons on the spot. No information could be drawn from her, as to the cause of this rash attempt. On the 27th October, her friends procured her a place, in the house of Mad. Fournier, but there, she still presented the same characters of melancholy and despondency.

On the 4th November, Harriet's mistress went out to walk, and desired the maid to prepare some dinner by a certain hour. She was also desired to procure some cheese at a fruiterer's shop in the neighbourhood. The woman who kept this shop had two children, one seven and the other nineteen months old. There existed between the mother of these children and Harriet Cornier neither intimacy, quarrel, or source of jealousy. When Harriet went to the shop at times, she always caressed and praised the elder child, named Fanny, and repeated these caresses on the day in question, with more than usual ardour, at the moment when, it was evident, she meditated the destruction of the innocent infant. The mother of Fanny informed Harriet that, as the weather was fine, she was going to take a walk with the child, and Harriet begged permission to amuse her while the mother put on some articles of dress for the intended promenade. This was complied with, though reluctantly, by the mother, who had no sooner gone up stairs than Cornier hastened home to her master's house with the child, and laying it on a bed, instantaneously severed its head from its body with a large kitchen knife! This bloody deed was done with such rapidity, that the infant had not time to utter a single cry! On a subsequent examination this infatuated creature declared that, during the murder, she felt no particular emotion—neither a sense of horror, of joy, or of fear. She confessed, however, that, a few minutes after the commission of this frightful act, she did feel a momentary remorse of conscience, which soon subsided. Leaving the decapitated corpse where it was, she first went into the bed-room of her mistress; but soon left that, and repaired to her own chamber, where she remained full two hours. By this time the mother of the infant arrived, demanding her child. Harriet coolly answered that "the child was dead." The mother at first thought that Harriet was in jest, but soon saw something in her countenance which struck a horror through her frame. She rushed past Cornier, and the beheaded corpse of her infant and the bed and floor deluged with blood, presented themselves to her view! At this moment Cornier snatched up the head of the murdered child, and tossed it through a window into the street. The father now came running to the house, and the first thing he saw was his child's head, which the wheel of a fiacre had just gone over in the street! All this time the murderer was coolly seated on a chair in the room near the body of the child, and making no attempt to escape. The commissary of police arriving, found her in a state of stupor. She denied no part of the act, but detailed all the circumstances—even the premeditation of the murder, and the arts which she had used to lull the suspicions of the mother, and enveigle from her the devoted victim of her bloody design. On being closely and repeatedly interrogated as to her motives for this terrible act, she either could or would not assign any other than that it was her destiny. When asked for her motive in hurling the head into the street—she answered that it was for the purpose of attracting public attention, and of drawing towards the house a sufficient number of witnesses to prove that she alone was the murderer. Among the spectators which soon collected on the spot, were several medical men, who declared it as their opinion at the inquest which was held, that Cornier was monomaniac, or insane on some particular point. She continued in a state of stupor, yet answered questions, though slowly, with precision and correctness. On the most minute investigation into her history, no trace of mental alienation could be detected, with the exception of the supposed attempt which Cornier had made to throw herself into the Seine. As a remarkable instance of the imperturbable state of the prisoner's mind, it was ascertained that the menses were flowing at the period of the assassination, and continued to do so, without the slightest interruption from the horrible act which she had just perpetrated, or the fear of any consequences which might result from thence.

In answer to interrogatories before a judge or magistrate, she averred that she was not ill—that she had no cause for chagrin or melancholy—that she had been married about seven years previously, but not happily so—that she attempted to drown herself because she was tired of changing her situation so often from one service to another—that the inclination to destroy the child came suddenly upon her, and that she never before had had such an inclination—that she experienced no particular emotion in perpetrating the deed, whether of gratification or repugnance—that she felt a momentary horror when the blood was flowing about—she was perfectly conscious of the nature of the act she was committing—that the fear of God did not deter her, because she then believed that there was no God—that she knew homicide deserved death—that she desired death, since life was to her insupportable. When asked finally if she persisted in acknowledging that she committed the murder, she answered steadily in the affirmative.

She was tried, for the first time, on the 27th February, 1826. She then appeared in a state of great nervous irritation—her limbs trembling—her eyes fixed—and her intellects in a state of stupor. A few days previously a medical commission, consisting of Drs. Esquirol, Adelon, and Léveillé, was deputed to examine into the moral and physical condition of Harriet Cornier, and after repeated investigations, collectively and separately, they reported their inability to detect any sign or proof of mental alienation in the prisoner. But they properly observed that, in many cases, it is extremely difficult to detect insanity—it requiring a long intimacy with the individuals, and numerous opportunities of watching them in all their varying states of temper and disposition. In fine, they reported that, although they were unable to adduce any proof of Cornier's insanity, they could not pronounce her to be free from any aberration of intellect.

This report being inconclusive, the advocate-general remanded the prisoner for another session. She was conducted to the Salpetriere, and there subjected to minute observation for three months, when M. Esquirol and his confreres made the following report:—namely, "that the most attentive observance of Cornier by themselves and the nurses could not detect any symptom of general or partial insanity. She was plunged in profound melancholy, but this might arise from the circumstances in which she was placed, and which she was perfectly capable of appreciating." But the doctors concluded their report with a wise reservation, that they could not vouch for her perfect sanity, especially if it was proved on trial that the melancholy, taciturnity, and other symptoms of a disturbed mind existed previously to the commission of the act for which she was now arraigned.

The prisoner was again put on her trial, the 24th June, 1826. She was seized with a fit of trembling on being led into court, and seemed to pay little or no attention to the act of accusation when read before her. Her answers to the interrogatories of the president were brief and in a low voice. Several witnesses deposed, that long before the commission of the crime, the prisoner had expressed herself as tired and disgusted with life—that she would sometimes laugh out loudly, and at others cry without any ostensible, or at least adequate cause. Several physicians were examined, among whom were Esquirol and Adelon, but they could not make up their minds to any certain conclusion, on the subject of Cornier's sanity or insanity. There was a great deal of reasoning and ingenuity adduced on both sides—the prosecution and defence—and the decision of the jury was, that Harriet Cornier had committed homicide *voluntarily*, but negatived the accusation of *premeditation*. The punishment was hard labour for life, and to be branded with the letters T. P. She heard the sentence without evincing the slightest degree of emotion.

We shall not follow M. Georget in his numerous remarks on this interesting trial. He properly criticises the advocate-general, who, in an elaborate speech for the prosecution, ridiculed the idea of a person being insane on a single point—in fact, he characterised the doctrine of monomania as a chimera—a modern resource for rescuing the guilty from the hands of justice—or an excuse for depriving arbitrarily a citizen of his liberty. It is useless to expend a line in refutation of this absurd assertion of M. Dupin. Unfortunately monomania is no chimera of the imagination—nor is it a modern disease. It existed in all ages; but formerly it was concealed under the denomination of melancholia, or hypochondriasis, of which, in fact, it is only a high degree. For this reason, there can be no doubt that thousands have suffered for crimes, or rather fatal actions committed during moments when reason had no dominion over the individual. We think our readers will agree with us that, although the physicians appointed to inspect the moral and physical condition of Harriet Cornier, could not detect any specific symptom or proof of insanity, yet reason and common sense, independently of any medical knowledge, would come to the conclusion, from the facts of the case, that this wretched female was insane at the time she committed the fatal act for which she was tried. We agree with M. Georget in the following sentiment:—"Un acte atroce, si contraire à la nature humaine, commis sans motif, sans intérêt, sans passion, opposé au caractere naturel d'un individu, est evidemment un acte de demence." The jury ought to have acquitted her on the plea of insanity —and the punishment should have been imprisonment, not hard labour, for life. We are convinced that this would have been the case had the prisoner been tried in an English court.

M. Georget coincides with Esquirol and many other physicians that the reports of these cases of suicide, homicide, and infanticide, tend greatly to produce repetitions of the same mournful scenes. Where there is any tendency to monomania, the recitals of such events accelerate the march of this disposition, and lead to explosions of a similar kind. In this respect police reports, and trials for murder and infanticide, have their disadvantages, as well as their advantages.

### 2. PARRICIDE.

He who doubts that *propensities* are born with us, should read attentively the following history. The mind is doubtless like a sheet of white paper *at birth*, as far as *knowledge* is concerned. No man comes into the world with a knowledge of his A. B. C.—but many are born with almost irresistible *propensities* to various actions or practices, good or evil, independently of the moral or physical circumstances in which they may happen to be placed.

The following case of Parricide happened at Metz. John Schmitt, the perpetrator of the horrible and unnatural act, was only 17 years of age, and it was clearly proved that, from his infancy, he evinced the most strenuous propensity to mischief, and even to ferocity. From the time he was able to run about the streets, he used to lie in wait, and amuse himself with throwing stones and filth at the passengers and even at animals that passed the house. Several people had thus been wounded, and as a child, John Schmitt got the name of the young madman. Happy had it been for himself and his parents, had he now been placed in some situation where he would either have been deprived of the power of doing mischief, or so severely corrected for his conduct as to have called forth his reasoning powers to prevent the penalty attached to such actions.

The sister-in-law of Schmitt, who resided in the same house, had caught the itch, and communicated it to the whole family. This caused great quarrels between her and Schmitt, which often came to actual blows, by one of which the female was severely wounded. On another occasion, he attempted to drown, and that not succeeding, to murder one of his cousins, Anthony Littre, aged 16 years. The latter was fishing with a rod and line on the bank of a pool, when Schmitt came to him and enticed him to a part of the bank which was higher, and where the water was deeper. Schmitt then suddenly pushed him into the pool, and mocked him when he cried out for assistance! The youth, however, got out of the water at another place, when Schmitt pretended that it was all in jest, and desired to know if the water had penetrated to the skin. Littre, to prove this, opened his waistcoat, when Schmitt instantly plunged a knife, which he had with him, into the boy's breast! Happily the wound was not mortal.

During the night of the 17th July, Schmitt's father was employed in boiling wood-ashes; and at 4 o'clock in the morning, called the son out of bed to assist him in taking the vessel off the fire. Young Schmitt came down in his shirt, and assisted his father in the operation above-mentioned; but while his father was stooping in doing something, the son seized a hatchet which lay on the floor and struck his father a blow on the head, which laid him prostrate and insensible on the floor! The wretch then ascended to the chamber where his sister-in-law and her husband slept, and wounded the former dreadfully by a cut of the same hatchet. This roused the brother-in-law, and the whole family, and Schmitt was secured. The father was raised up and put to bed, but almost immediately expired! Schmitt profited by a moment's liberty which he got, to huddle on his clothes, and made an attempt to spring through a window into the street, but was prevented. He then demanded to see his father. When conducted to the bed where the corpse lay, he raised the sheet off his father's face, and pronounced these remarkable words:—"Ah! my dear father, where are you now—and where shall I soon be! It was yourself and my mother who were the cause of this misfortune, and long ago did I predict this to you! If you had brought me up better, this would never have happened."

When interrogated as to the motive which urged him to the horrid deed, he replied that it was unquestionably the Devil. He also pleaded that the itch which had been communicated to him by his sister-in-law, and which had been driven in by some application, was the cause of occasional loss of reason, and of violent and ungovernable impulses to immolate every thing that came in his way. Numerous witnesses proved that this boy exhibited, at times, the most profound sense of religion and piety—such was the strange compound of opposite propensities in this youth.

M. Vivier, the advocate who defended him on his trial, made a most talented speech in favour of Schmitt's insanity, during which, he was often interrupted by the prisoner, who declared that he was not insane. The jury, after ten minutes' deliberation, brought in a verdict of guilty on all the counts of the declaration. He was, therefore, condemned to death.

To the counsel who had been granted him for his defence, Schmitt confessed that, whenever an instrument of any kind came into his sight, he felt the most urgent desire to wound or kill the first person that came in his way.

After the trial, he refused to petition for mitigation of punishment, urging as the cause of this refusal, his wish to spare his mother the wretched feelings of suspense, during the delay which this petition would occasion—being thoroughly convinced that there was no mercy for the PARRICIDE. On the night before his execution, when presented some victuals, he asked what it was o'clock; and being answered that it was very near midnight, he refused the food, observing that, in a few minutes more, it would be *Friday* morning, when it would be a sin to eat meat! When walking barefooted to the scaffold, he was asked by his confessor if the stones did not hurt his feet, (which, by the way, appears to have been a very idle question of the holy father,) he replied in the negative; and wished that they had condemned him to walk over thorns in his path to the grave! On the scaffold, one of his hands was first cut off; during which he evinced not the slightest suffering, and never uttered a complaint up to the moment when his head was severed from his body.

M. Georget had seen this unfortunate youth many times, and was always struck with the extremely diminutive size of his head, as compared with his body, and also with its unusual configuration. His forehead was exceedingly low and narrow—the sinciput was very much elevated—and, above each meatus auditorius, was a very remarkable prominence. The skull of this individual presented a great resemblance to those described by M. Pinel as belonging to idiots.

"Observe," says M. Georget, "in this wretched youth, the strange assemblage of religious and wicked propensities—two kinds of sentiments which would seem to repel each other, and the re-union of which, in any one individual, would seem impossible, except in consequence of the peculiar organization of this young man's head. Observe the horrible acts which Schmitt committed, without any ostensible motives, and at so tender an age—consider the exclamation which he uttered on viewing the corpse of his father—the avowal which he made, that he was always haunted with the propensity to shed blood—contemplate the formation of his head—and you will be constrained to admit that this youth was disgraced by the hands of Nature from the moment of his birth—and that he brought into the world with him those vicious propensities which had not been sufficiently counter-balanced by ideas of morality, justice, and the fear of punishment."

We do not see how these sentiments can be negatived. They are favourable, it is true, to the doctrines of phrenology; but not in repugnance to reason, religion, and morality. These vicious propensities, though they might not be eradicated, might be greatly coerced by proper education, and by proper discipline. Had Schmitt been severely chastised, early in youth, for each of those mischievous tricks which he performed, the fear of punishment would have ultimately counteracted the propensity to misdemeanour. How do we break the spirit and curb the bad qualities of an unruly horse, when young?—By education and discipline truly! Had Schmitt been confined to hard labour or solitary captivity for a proper period after his first attempt to drown and murder his cousin, he would not have committed parricide. We see nothing in the doctrine of *organic propensity* to clash with the very best principles of education, moral and religious—but every thing to strengthen the propriety of early discipline, early coercion of the passions, and early chastisement for every deviation from the paths of rectitude.

In respect to the individual, whose history and ignominious death we have recorded, M. Georget gives no opinion, as to the justice or injustice of his sentence, unless his sentiments can be gathered from the passage which we have literally translated above. We think no man can aver that Schmitt was in a state of perfect sanity of mind. But it would be also difficult, if not dangerous, to aver that he was not amenable for the act which he committed, though not of sound mind. There may be culpable homicide, even in mania. It is quite evident that Schmitt could never again have been safely let loose upon society; but his history and fate offer a lesson to parents not to neglect the correction of early vicious propensities.

### MISCELLANEOUS CASES.

3. *Case of Mons. Tristel.* In January, 1821, there was to have been executed at Rouen, a young man of 17 or 18 years of age, belonging to a respectable family in easy circumstances, for the crime of attempting to poison several of his relations:—but he anticipated the executioner by putting himself to death by the same means. Dr. Vingtrinier, surgeon of the prison of Rouen, examined this young man's body, and after investigating the immediate cause of death—arsenic taken into the stomach—proceeded to dissect the brain, and there he found unequivocal evidence of chronic inflammation of the membranes, of considerable standing. The following were some of the principal facts which came out on Tristel's trial. *First,* the prisoner had experienced, in his infancy, great pains in his head, and much tendency to somnolency—2d, at the age of ten years, he became triste and melancholy, preferring solitude to society, and evincing a strong propensity to torment animals, and break and destroy all the little articles or objects of amusement belonging to his comrades. At the age of puberty, his intellectual faculties, were but imperfectly developed; and it was observed that he was very much in the habit of calculating sums of money, and employing himself in speculations which shewed that he entertained a strong inclination to amass riches. It appeared also, that this dominant idea engendered a secret jealousy towards his brothers and sisters—and ultimately the horrible design of poisoning not only them, but also his parents! The family were numerous, and many of his brothers and sisters married and living at a distance. Some of them also had families of their own, so that to make himself the sole heir of the paternal property, he saw it necessary to destroy a great number of individuals—

at least fifteen or twenty! In the year 1820, he contrived a reunion of almost the whole family circle at his father's house, with the intent to poison the whole! Some days previously, he procured, at a chemist's shop, a large quantity of arsenic, under some pretence or other, which he distributed, the day of the banquet, in the soup and other dishes which were preparing for dinner. The quantity of arsenic thus distributed, was so great as to cause instant vomitings in those who partook of the victuals, in consequence of which, no person died of this horrible attempt.

It is properly supposed by both Dr. Vingtrinier, and M. Georget, that such a horrible and *extravagant* project could not have been engendered in a sane mind—and the appearances, on dissection, proved almost to a demonstration, the insanity of the individual. His condemnation therefore to death, though naturally to be expected from a jury who could not comprehend the nature and effects of monomania, was probably not consistent with strict justice.

4. *Case of Matricide.* At Bergerae, in the course of March 1826, a strange and horrible matricide was committed by a young man of good education, and who was greatly attached to an aged and infirm mother, whom he attended and nursed with the most filial solicitude. The parent being threatened with death, by some acute disease, the son became greatly agitated, and plunged into profound grief. He fasted and prayed without ceasing, for the recovery of his parent. On the day before the commission of the dreadful act he appeared more tranquil than usual. In the evening he prayed in the presence of his mother and the servants, and went to bed without evincing the slightest aberration of intellect. He had not been more than a quarter of an hour, however, in bed, before he started up and repaired to his mother's chamber, where he announced to her, with apparent transports of joy, that he was an angel commissioned by God to deliver her from all her sufferings. He had scarcely pronounced these words, when he seized his parent by the throat, dragged her out of bed, and quickly put a period to her existence by repeated blows with a solid chair which was in the room! The unfortunate wretch was seized and put into prison; but it required four men to guard him night and day, in order to prevent his destroying his own life, by dashing himself against the walls and floor of the gaol. The adjudication of this case is *not given*, but we suppose no jury would think of bringing in any other verdict than that of insanity. Here is an instance of *moral* circumstances overcoming the understanding, and producing the same effect as physical lesion of the brain. It is a very different thing, however, from that propensity to evil which comes along with existence, and seems to depend on organization. The youth who was thus deprived of reason by a too anxious filial solicitude, stands in a very different light from the youth who committed parricide from unrestrained indulgence of vicious propensities. The latter might perhaps deserve mercy from a tribunal of justice—but the former is entitled to commiseration and sympathy.

It appears that the tribunals of justice and the crown lawyers in particular of France, are averse to the admission of such a state as homicidal, suicidal, or infanticidal monomania, and the various trials of individuals seem to authorise this conviction of our continental neighbours. M. Georget raises his voice against this injustice in the criminal code of his country, and not without considerable effect. He justly observes that the monomaniac may preserve a very fair portion of intellect on every other subject but that which is directly connected with his hallucination. The dominant idea may also change, and vary its object. If you disperse one chimera from the mind, another rises, and these exclusive illusions may thus succeed each other *ad infinitum.* On this account it is very difficult sometimes to determine whether such and such an act has reference to the mental illusion that, at the moment, possessed the individual.

Again:—The melancholic insane, or the monomaniac, may preserve a profound silence, *for years,* on the subject of their hallucination. Pinel mentions the following fact:—A commissioner went to the Bicêtre, for the purpose of liberating those whom he considered cured. He particularly interrogated an old vine-dresser, who evinced not the smallest symptom of mental alienation during a minute examination. When the procés-verbal, however, was delivered to the peasant for his signature, before being discharged, he very coolly signed himself JESUS CHRIST, and then broke off into ridiculous asseverations respecting his own divine character.

5. *Case of Homicide.* On the 12th February, C. E. N. aged 30 years, murdered an infant of four years of age, (near the gates of Konigsberg) with whom she had travelled in a voiture from one of the neighbouring villages. The culprit continued to draw the conductor of the voiture a little aside, and then with a large knife which she had concealed about her person since the preceding day, she severed completely the child's head from her body! The conductor was the father of this child, whom she persuaded to bring with him on the journey. This female immediately confessed the act, and delivered herself up to public justice. She then declared, and always after kept to the following story. She had had a quarrel with a neighbouring female, by whom she was cited before a magistrate, who threatened a farther prosecution at the ensuing sessions. In order to avoid this disgrace, she took to flight, and wandered about for some days, without any fixed design or resolution. At length she came to the house of a peasant, with whom her brother had some acquaintance, and there she sojourned for some time, under various excuses, but without betraying the least symptom of melancholy. It was in this situation that she suddenly formed the resolution of assassinating her hostess's child, notwithstanding the many marks of hospitality and kindness which she had received at her hands. This was the infant which she murdered, and the following was the only reason she could offer for the sanguinary act. "The peasant's daughter was an *only child*—so was she herself an only child—and yet she was always very unhappy and unfortunate:—The child would most likely be so too, and, therefore she determined to take its life."

It came out in evidence on the trial, that this woman had, more than once during the preceding two years, exhibited symptoms of mental derangement, and means were taken to place her under restraint, but she had evaded this by flight. Afterwards she became apparently well, though somewhat reserved, and continued so up to the time of the citation before the magistrate, as above detailed. In a medical consultation, the prisoner was considered as guilty of murder, because she had shewn a *premeditation* in the affair, and, consequently, that she was not protected by insanity, which they admitted to exist. The judges, however, were of a different opinion, and ordered her to be confined as a maniac. This happened in the year 1788, and it appears that a revolution has since taken place; for the medical profession, in France, have come to the proper way of thinking, while the judges and lawyers have reverted back to the less enlightened path of the Konigsberg physicians. It is clear, we think, that in this case, there was a *premeditation* founded on an *insane notion*, which greatly alters the case from a premeditation in a sane mind. Upon the whole, we consider the judges were right in their decision, though certainly it was a very nice point to decide.

6. *Case of Homicide.* We shall conclude this article on a melancholy but interesting subject, with the following particulars of a recent homicide.

On the 20th May, 1825, Josephine Durand, a servant of the Sieur Mailfert, left her master's house early in the morning, and did not return till late at night. She was then asked if she had found another situation, which they supposed she had been seeking? She replied in the affirmative, and that the place would be a lasting one for her! All the evening she appeared greatly agitated, and next morning, on some questions being asked by her mistress, she confessed that she had murdered an infant the preceding evening. In a short time an alarm was spread that a child in the neighbourhood had disappeared. Josephine then set out, as she said, to deliver herself up to the magistrate. It appeared in evidence, as well as by the prisoner's confession, that the child, six years of age, had given its hand to the prisoner, who directed her course towards the fields, and although they were met by several persons, Josephine had always taken the precaution to guard her face from being seen. After some research, the child was found strangled in a field of rye, and covered over with a white table-cloth, which was proved to belong to the mistress of Josephine. She was therefore accused of the murder, and confessed the act.

It was proved that the prisoner not only strangled the child, but took from the person of the deceased some gold ornaments which she wore. This also the prisoner confessed. It was ascertained (from the prisoner's own confession) that she had kept the child full an hour in the rye-field, before she had strangled her. Josephine was therefore put upon her trial for the double crime of murder and theft. The prisoner, who was 23 years of age, heard the accusation read, without exhibiting any particular emotion, and replied to all interrogatories with clearness and precision. Several witnesses, however, proved that the prisoner had been subject to epileptic or rather hysterical attacks, and that she was thought sometimes to be rather deranged in mind. This last part was not clearly substantiated. The advocate-general charged the jury to find the prisoner guilty, as there was no proof of insanity, and as it would be opening a dangerous door to guilt, if they acquitted her of such a crime, under the circumstances which came out on the trial. Her counsel, M. Talon, made a most admirable and ingenious defence, and, we have no doubt, saved the young woman's life. The advocate made a powerful appeal to the feelings of Frenchmen, by asking them if they would place a stain on their country, by believing that France could produce such a monster as Josephine appeared to be, if she committed the act in her proper senses. This last appeal seems to have turned the scale in favour of the prisoner. The jury found Josephine guilty of committing homicide on the person of Victorine Houille—"but involuntarily and without premeditation"—"mais *sans volonté* et *sans premeditation*." That the homicide had been accompanied by abduction of property ("soustraction") but *not fraudulent* abduction; and that the act was not committed on the public high-way.

By this verdict, the prisoner was acquitted of the accusation; but the minister ordered the prisoner to be detained in a place of safety, till the state of her mind could be satisfactorily ascertained.

M. Georget thinks that Josephine was in a state of mental imbecility, rather than insanity, in consequence of which, she had not sufficiently distinct notions of right and wrong. He believes that she committed the homicide for the purpose of effecting the theft, which, under other circumstances, would aggravate the crime. "In confining this individual," says he, "for life, the public safety was sufficiently secured—to have put her to death, would not have prevented similar acts of homicide on the part of others."

The verdict was singularly merciful, compared with some of those which we have here put on record, and where insanity was unequivocal. Perhaps, however, it was erring (if erring) on the right side. Seclusion for life is punishment enough for almost any crime, and more severe, we imagine, than death, while it is (however paradoxical) more humane. It secures the public from any danger, as far as the individual is concerned—it gives the prisoner time for meditation and repentance—and prevents those horrified feelings inseparable from public executions, and which we verily believe, tend to harden the guilty rather than prevent guilt.

---

### 12. CHRONIC ULCER IN THE STOMACH.

We have lately recorded some instances of chronic ulcers in the stomach, unattended by any well-marked symptoms, till the bottom of the ulcer gave way, and permitted extravasation of the gastric contents.

Mr. Hunter, of Mincing Lane, has lately added another case to the list, as published in the January number of the Medical and Physical Journal.

A married female, so corpulent that she thought herself dropsical, and who appears to have been thought so by her physician—had complained of pain occasionally at the navel, for four years previously. This, however, did not prevent her attendance to her domestic affairs, and her spirits were good, as was her appetite. On the 16th August, 1826, after eating a very hearty dinner, she was suddenly taken ill with severe pain in the umbilical region, attended with exquisite tenderness of the part. The pulse was but little affected. After taking 30 drops of laudanum she vomited a quart of fluids and half-digested substances, but without any material relief. Opium in considerable quantities, venesection, blisters and other remedies, were employed in vain, and she expired in 30 hours from the attack.

On dissection, an ulcer was found near the lesser curvature of the stomach, about two-thirds of an inch in diameter, and partly surrounded internally with thickened, white, and shining edges. This had opened, and permitted the contents of the stomach to pass out into the abdomen, thereby occasioning the fatal catastrophe. The heart, as well as other parts of the body, was loaded with fat.

It is not a little remarkable that ulcers will continue for years in the stomach occasioning but little disturbance, comparatively speaking; while in other individuals, the most trifling irritation of that organ will throw the whole system into confusion.

---

### 13. DYSMENORRHŒA.

Dr. William Campbell has favoured the public with an elaborate dissertation on this complaint, in the January number of the Edinburgh Journal of Medical Science, in which considerable erudition and research are evinced. On this, as on many other occasions, the conflicting opinions of ancients and moderns do not much elucidate either the pathology or treatment of the complaint, and the doctor's own observations, contained in one or two pages at the end, are worth the whole of the researches that occupy so many pages in the beginning. For our own parts, we entertain so much respect for Dame Nature, that we are not very fond of officiously interfering in trifling, or even considerable deviations from the ordinary course of menstruation, unless it appears that the general health, or the patient's own feelings are suffering severely from the irregularity. Dysmenorrhœa, and even emansio mensium, are more frequently the consequences than the causes of bad health. But this is not a popular doctrine, either in or out of the profession, as the following passage will shew.

" In 1822, a young lady, 19 years of age, of tall stature, healthy constitution, and void of every complaint, except a voracious appetite, was, by the advice of a surgeon in high estimation, *first*, heartily ducked once daily, on the sea-beach for a summer season to strengthen her—

*secondly*, stewed in a warm bath, *for several months*, to relax her—and *thirdly*, made to swallow for some time daily, such a proportion of carbonate of iron, as would almost have led one to think that she was compelled to use it as a substitute for oatmeal. Why all this professional purgatory? Because the lady's catamenia had not made their appearance! To shew that the lady had an excellent constitution, and could therefore require no medicines, she withstood those *violent attempts on her person*, without any injury to her health."

It must be confessed, however, that medical men are often so importuned by mothers and nurses, as to be forced to prescribe where they had rather let nature alone. In such cases, they should avoid heroic remedies.

Dr. C. observes that our indications, in dysmenorrhœa, are, *first*, to diminish pain and local irritation during the presence of the catamenia: and *secondly*, to adopt means, during the intervals of menstruation, for the removal of that condition of the uterine or general system on which dysmenorrhœa depends. For the first object, Dr. C. recommends that from four to six leeches should be applied to each groin, on the immediate approach of pain, and the bleeding promoted by warm fomentations. Warm injections per vaginam and the hip-bath are serviceable, though the former is seldom complied with, in this country. In respect to opium and hyosciamus, Dr. C. avers that the former is sometimes hurtful, and the latter inert and useless. He has found a full dose (ʒss.) of camphor, very effectual; especially if aided by injections of assafœtida. When the catamenia begin to cease, warm injections, *per vaginam*, are very serviceable. In full plethoric habits, where the catamenia are sufficient in quantity, but attended with pain and spasm, warm cloathing, the hip-bath, quietude, and two or three doses of a few grains of calomel at night, with phosphate of soda in the morning, should precede the period. Sexual intercourse should be forbidden, where the dysmenorrhœa is produced by unusual irritation of the uterine system.

In cases of debility, or torpor of the uterine system, as it is called, Dr. C. has never obtained *much* advantage from the mineral tonics or stimuli. He speaks more favourably of the vegetable world—as the colombo, gentian, quassia, or cinchona, with a moderate proportion of claret or madeira, and a proper degree of exercise, as riding and dancing —"particularly that form of it termed *waltzing*." We think certain gymnastic exercises would be more convenient as well as more effectual in such cases. Electricity, sulphureous waters, aloetic aperients, &c. are occasionally beneficial in these states.

The ergot of rye has been exhibited by Dr. C. in three cases. In one of these, the patient was a widow, who had aborted fourteen or fifteen years previously, and, ever since that accident, her catamenia had been very sparing, and secreted with great pain, although regularly as to time. She was in the habit of passing "substances of a cutaneous texture," during the catamenial nisus. The uterus was scirrhous. In one of these painful periods the ergot was administered (ʒj. boiled in four ounces of water to two ounces) in decoction. The pains were slightly mitigated. She took half an ounce of this decoction daily, during the next interval, and then menstruated without any pain. The results of the other two cases are not stated. The ergot is deserving of further trial.

### 14. EXTRA-UTERINE FŒTUS.

Mr. Mackie, surgeon, Demerary, has communicated to us the particulars of a very curious case of this kind, the full details of which will be found in our contemporary, the Medical and Physical Journal, of this month.

On the 9th October, 1826, a negress, 40 years of age, was admitted into the Lying-in-Hospital of the Plantation, Richmond Hill, Leguan Island, for confinement of her twelfth child. Next day, the medical attendant of the estate was summoned, as the nurse thought the labour was going on slow. On examination, per vaginam, he concluded the labour had not yet commenced, as no change in the parts had taken place. The 11th, found the patient with slight labour pains, and she complained of numbness in the lower extremities. The head of the child could be felt distinctly resting on the pubes—the os tincæ could not be touched by the finger. The patient was otherwise in good health and spirits. At 7 in the evening of this day, while the patient was in a half-sitting half-reclining posture, her extremities became suddenly cold, and, without complaining of any pain, she expired in a slight convulsion.

An incision was immediately made from the umbilicus downwards to the pubes, when a full-grown male fœtus, with its umbilical cord and placenta, was protruded with considerable force. The child was perfect in every part, and was of rather a large size. It was not inclosed in any membrane or sac, nor could any connexion be traced between the fœtus or placenta and any of the abdominal viscera! The uterus was in its natural position, and about the size of that organ in the third month of pregnancy. Not the slightest trace of rupture of the womb could be discovered. Both the fœtus and the uterus are preserved, and now in the possession of Mr. Mackie.

A more rigid scrutiny would have discovered, we should imagine, the place where the placenta was attached; but even so, the case is a very curious one, and may furnish food for reflexion.

### 15. M. PIORRY'S PLEXIMETRE.

M. Piorry has announced that, by means of a small pallet of ivory placed on any part of the abdomen or thorax, he has been enabled to turn percussion to a much better account than has yet been done; the sound emitted by the portions thus covered by the ivory pallet, being much clearer and more indicative of the actual state of the parts underneath, than when these parts are stricken by the fingers in the usual way. M. Piorry has made more than 80 experiments on the living and dead subject, healthy and diseased. By means of this intervention, sound may be elicited from the thorax, even when the integuments are œdematous. The extent of pleuritic effusions may be ascertained with great exactness. Hepatisation, and also a tuberculated condition of the lungs can be more readily detected in this way than by simple percussion. But it is in the abdominal region that the pleximetre promises to be of most service. The extent which any solid organ, as the liver, occupies —that of tumours—watery effusions, &c. may be easily ascertained by the difference of sound on percussion of the tablet instead of the naked parietes. Even the quantity of fluid in the stomach or intestines can be appreciated. The state of the uterus (when pregnant) can be also correctly estimated through the medium of the pleximetre.

If discoveries go on in this manner for half a century more, there will be little occasion for the ancient desideratum—a glass window in the breast—to see what is going on within.

---

### 16. INTESTINAL ULCERATION AND HÆMORRHAGE.

For many years past we have been anxiously directing the attention of our brethren to the state of the intestinal canal in fever. We have had but too many opportunities of observing how superficially the examination of bodies is conducted, especially in regard to the mucous membranes—and how often the diseases of these parts are overlooked. The profession is much indebted to Dr. Hewett, of St. George's Hospital, a most able, zealous, and liberal minded physician, for directing the attention of practitioners in this country, to the state of the gastro-intestinal mucous membrane in fever. We are convinced that the more minutely the dissections, in fatal cases of fever, are performed, the more frequently will Dr. Hewett's observations be found correct. We shall now relate some particulars of an interesting case which lately came under our observation, in illustration of these preliminary remarks.

A fine young man, a Law Student, aged about twenty one years, became affected with common fever in the beginning of December, 1826, and was under the care of Mr. Bampfield of Bedford-street, Covent-garden. The fever ran on for more than a month; but convalescence had at length apparently commenced, and the patient's appetite was returning, without any obvious organic affection having been produced by the protracted fever. About the end of the first week in January, 1827, and without any apparent cause, a sudden and extensive hæmorrhage from the bowels took place, and this continued to recur daily for eight days in succession, when the writer of this article was called into consultation. On examination, the pulse was found to be from 90 to 100, and rather weak—the tongue slightly furred in the middle, but red at the tip and sides—skin of natural temperature, and without perspiration—mental faculties clear—respiration natural. The abdomen was carefully examined. There was slight tenderness in some places, but no distinct pain. The abdominal muscles, however, were always tense, whenever the hand was placed on the belly. He had passed in the preceding 24 hours about two pints of blood, without any coagula, mixed with watery fluids, but without any fæcal matters or smell. No real fæculence was seen by Mr. Bampfield in the stools for the preceding week; and the patient lost at least 20 or 24 ounces of blood daily during that period. Superacetate of lead and opium were given for three or four times, and then a gentle aperient. This made little alteration in the discharge. Two half-drachm doses of oil of turpentine were then given. The first dose came up, the second was retained. For two days, the blood disappeared from the stools, and some traces of fæculence were observed in the liquid motions. The hæmorrhage then returned. After exhibiting various remedies, the hæmorrhage again stopped, or nearly so, for three days, and decided proofs of fæculence were in the motions. It was now fondly hoped that the danger was over; but in the midst of these promising appearances, the patient was suddenly seized with a great prostration of strength, and died in the course of a few hours—about 16 days from the commencement of the intestinal hæmorrhage.

The suddenness of the death induced the writer to suppose that ulceration had existed in some part of the intestinal canal, and that perforation of the bowel had taken place, with extravasation of fæcal matters into the general cavity. Part of this prognostication was true, and part was false. On examination, in presence of Mr. Brookes, surgeon, and Mr. Owen, his assistant, a series of ulcerations were found in the ileum, varying in extent from the size of a split pea to that of a half-crown piece,—and from a slight abrasion of the mucous membrane, to complete destruction of all the coats, except merely a pellicle of peritoneum, through which boundary the ulceration had not passed. The ulcers gradually increased in size and depth as the ileo-cæcal valve was approached, where they occupied nearly the whole circle of gut. There was no ulceration above or below the intestinum ileum.

Now we need hardly say that these ulcerations were formed during the fever, and that from these the hæmorrhage proceeded, when the fever was, in fact, over, and convalescence was apparently commencing. Yet here had been no diarrhœa—no pain complained of in the abdomen,—and scarcely any tenderness on pressure, up to the very day of this patient's death. Had the hæmorrhage not taken place, and the patient died, who would have suspected intestinal ulceration?—very few we believe. The case offers an instructive lesson—namely, that gastro-enteritic inflammation, and even ulceration, may be going on in fever, without any obvious or well-marked symptom to indicate its existence. A superficial examination of the abdomen, after death, without opening the intestinal canal itself, would not have revealed the true state of things—for in many places where there was serous ulceration of the mucous membrane, there was little alteration in the appearance of the external surface. We hope this example will induce practitioners to suspect the existence of gastro-enteritis in fevers, where the symptoms are equivocal—and to carefully examine the *internal*

surface of the alimentary canal where an opportunity of dissection is given.†

The case was attended, during the latter days of the patient's life, by Mr. Brookes (Mr. Bampfield's successor) in company with the reporter.

---

### 17. EXTRACT OF BALS. COPAIBÆ IN GONORRHŒA.*

Surgeons are well aware that the balsam of copaiba would be often successful in gonorrhœa, if they could prevail on their patients to continue its use for a certain time. But this they rarely can do, from the nauseous taste of the medicine, and its effects on the stomach and bowels. It would be extremely desirable that the article could be deprived of its disagreeable qualities, without injury to its medicinal; and this Mr. Thorn thinks he has done by the preparation of a resinous extract, from which the essential oil, possessing the obnoxious part of the balsam, is separated. The remaining extract is supposed to contain "all the virtues of the copaiba."

Mr. Thorn divides gonorrhœa into three stages—1st. that which occurs within twenty-four hours of the first appearance of the symptoms:—the 2d, is when the inflammatory symptoms are fully established—and the 3d is, when the inflammation has ceased, the membrane of the urethra being in a state of atony, and the only symptom being a thin mucous discharge.

Medical men have generally been afraid to exhibit the bals. copaib. in the second, or inflammatory stage, and the first stage has often elapsed before they are consulted. It is in the first two stages, that the extract of copaiba has been found most advantageous. Mr. Thorn has related one case where the disease was cured in a couple of days, when the medicine was given in doses of fifteen grains to a scruple thrice daily. In the inflammatory stage, unless there were symptoms indicative of approaching swelled testicle, he has not hesitated to give the extract in doses of ten or fifteen grains thrice a day. In no case has he found it produce any other effect than that of relieving the scalding, and frequently doing away with this symptom, in a short time. The medicine should, of course, be continued for a few days after all the symptoms are gone. Mr. Thorn very properly combines the depletory treatment and cooling regimen with the exhibition of the medicine.

In the third stage, that of gleet, too often dependent on some alteration of structure in the mucous membrane of the urethra, the extract of copaiba has not proved so useful. Our author speaks favourably of an injection composed of one drachm of extractum tormentillæ to six ounces of water. It has been used with advantage by Mr. Tyrrell, of

---

† We beg the reader to refer to the first article in this Number, page 327, proposition 136, for an explanation of some remarkable phenomena in the above case, especially the want of pain on pressure of the abdomen.—*Ed.*

* Mr. Thorn's Observations on the Treatment of Gonorrhœa, 8vo. pp. 57. 1827.

St. Thomas's Hospital, and also by Mr. Alcock. Several cases have been communicated to our author, and are here published, from Mr. Tyrrel, corroborating the efficacy of the extract of copaiba in gonorrhœa. The medicine is prepared by Philp and Co. 62. Quadrant, Regent-street. We think the surgical profession are indebted to Mr. Thorn for thus early and candidly communicating the results of his experience with the extract of copaiba.

---

### 18. CARBONATE OF IRON IN NEURALGIC AFFECTIONS.

This preparation is daily increasing in fame, and we are convinced that it will prove one of the most valuable medicines in the pharmacopœia, for very many of those anomalous and painful affections of nerves, where no vascular excitement obtains. Two cases are reported in No. 174 of the LANCET, from the hospital practice of Dr. Elliotson, of which we shall take a very concise notice.

1. The first was that of a female, 33 years of age, who had suffered for three months, with a violent pain in one of her legs, commencing in the great toe, running along the inside of the tibia to the ham, the groin, and across the lower part of the abdomen round to the loins. It came on rapidly, especially by pressure or friction, and was of a shooting or stabbing kind. She was ordered the carbonate of iron, *two drachms* every six hours. In five days the pain was relieved, and the medicine was continued for a fortnight longer, when the improvement not being progressive, she was ordered *half an ounce* thrice a day. In three weeks more she was reported, "quite well."

2. The second case was rather more puzzling, and presented some of the phenomena of angina pectoris. The patient was also a female, 36 years of age, who had suffered for seven months with violent pain in the left side of the chest, occurring at intervals, and shooting from the region of the heart over the left breast, and sometimes down the arm, and backwards towards the spine, During the paroxysm she experienced dyspnœa, amounting to a sense of suffocation. Pressure or friction brought on the attacks, or even a deep inspiration; but the pain was never brought on by quick walking. The pulse was regular, and the stethoscope detected no organic disease of the heart. For two months she was bled, blistered, cupped, and took digitalis, colchicum, and prussic acid, without benefit. She was then ordered to take half an ounce of the carbonate of iron thrice a day, and in little more than a fortnight she was cured.

The symptoms of real angina pectoris *generally* depend either on ossification of the coronary arteries, imperfection of the valves of the heart, or on a peculiar softening of the muscular structure of the organ. In none of these cases have we ever seen much good, but frequently mischief, result from depletion or the use of sedative medicines, as digitalis, colchicum, &c. with the exception of opium. In the pseudo-angina pectoris, like the instance above detailed, we cannot expect benefit from depletion; and we much wonder that so talented a physician as Dr. Elliotson should have persevered two months in such unfeasible measures, especially when he ascertained by the stethoscope that there was no organic disease of the heart. In these imitative cases, there is usually some nervous irritation, and a morbid susceptibility in some portion of the alimentary canal, and we conceive that the carbonate of iron removes this susceptibility by its tonic effects on the sentient extremities of the gastro-intestinal nerves. This susceptibility often returns, if the digestive function is not regulated, and the secretions kept in a good state.

---

### 19. GENERAL VASCULAR INFLAMMATION.

Considerable doubts have been lately thrown on the nature of those appearances in certain structures of the body (especially the internal surfaces of vessels and hollow viscera) which have currently passed as proofs of inflammation having existed during life. These doubts may be carried too far, and it is necessary for us to be on our guard against errors on either side of the question. The following cases communicated by Dr. Dommanget to the Societé de Medecine, are not without considerable interest.

*Case* 1. Francis Blanc, aged 25 years, a French soldier in the Madrid Garrison, was brought into the Military Hospital on the 20th September, 1823, having been ill for a fortnight in the neighbourhood of the Spanish capital. The pulse was strong and frequent—tongue red—great thirst—face suffused—conjunctivæ injected—breathing precipitous and impeded—dry cough. On percussion, the chest sounded very dull—there was a burning heat at the epigastrium, and indeed over most parts of the body. Venesection to *eight ounces*—emulsions—cold applications to the abdomen, and warm fomentations to the chest. 21st. The thoracic symptoms were diminished—those of the abdomen continued. Thirty-six leeches to the epigastrium. In the evening something better—tongue less red—frequency of pulse abated. 22d. The patient was better; but during some devotional exertions, he became suddenly worse. The gastro-intestinal phlegmasia increased rapidly, accompanied by sympathetic affection of the head, rising to delirium, requiring some men to confine the patient in bed. The tongue got dry—the thirst inextinguishable—the eyes sparkling—delirium loquax. 23d. Same condition. Thirty leeches to the epigastrium and tract of the jugulars. Ice to the head—warm fomentations to the abdomen. 24th. No alterations in the state of the pulse. In the evening the patient got some wine clandestinely, immediately after which the delirium was greatly exasperated, and a strait-waistcoat was applied. 25th. No material change. Forty leeches were applied to the epigastrium and neck—cold to the head continued. 26th. The patient is more calm; but the force of the pulse is nowise abated. He passed black blood by stool, in some quantity. 27th and 28th. Same state continued. 29th. The delirium is now changed to coma, with a kind of periodical trismus. The pulse is still unabated. Colliquative diarrhœa added to the other symptoms. In this deplorable condition the unfortunate patient continued two days, and then expired.

*Dissection.* There was some effusion between the membranes—opacity and thickening of the arachnoid—softening of the cortical substance of the brain—water in the ventricles. The same conditions were observable in the spinal canal, which was entirely opened. The lungs appeared sound exteriorly, but the parenchymatous structure shewed many points of chronic inflammation, and some crude tubercles. The mucous membrane of the lungs was extensively inflamed, and the bronchia filled with viscid mucus. The pericardium adhered in some places to the pleura of the lungs. The heart was pale, and its structure flaccid. "The ventricles and auricles were evidently inflamed, as were the mitral and tricuspid valves." The arteries, at their origin from the heart, were of a cherry-red colour, which extended, in the pulmonary artery, to its divisions and subdivisions—in the aorta, to its iliac bifurcation. The carotids, jugulars, and subclavian vessels were all inflamed—and the appearances of inflammation could not be washed off by repeated lavations. There were several marks of phlogosis in the mucous membrane of the stomach and bowels. Near the cæcum there were ulcerations to a great extent, with tumefaction of the mesenteric glands. Some of these were as large as pigeon's eggs, and degenerated into a melanose structure. In the colon were numerous marks of inflammation—several vestiges of cicatrised ulcers—and many ulcerations still open. There were several other morbid appearances in the body, but none of them apparently connected with the immediate death of the patient. There were, however, marks of inflammatory action in almost every part of the body—even in the joints, absorbent glands, &c.

*Remarks.* Our author has never seen or read of an instance presenting proofs of such universal inflammation, as this case afforded. Perhaps indeed, if examinations were made more minutely, there would be found more general tokens of a phlogosed state in those who die of diseases with great excitement. The interior surfaces of the vessels—the spinal marrow—the ganglia—the glandular system, are but rarely examined, on account of the time necessary for such investigations. In the present case, our author thinks he might be justified in concluding that there was a general phlogistic diathesis; but he considers it more correct to conclude that the inflammation began in one organ or class of parts, and involved others in succession. "It appears to me that the inflammatory process commenced in the mucous membrane of the digestive tube and respiratory apparatus, and was thence propagated by sympathetic association, to the heart and the brain—and finally that, from these last centres of vitality, the phlogosis was extended to the various structures dependent on them, or connected with them."

*Case* 2. A fusileer in the eighth regiment of Swiss Guards, aged 23 years, entered the Military Hospital of Madrid on the 31st of January, and died on the 8th of March. The following were the principal phenomena presented to the observation of Dr. Antonini, who was then in charge of the Military Hospital. When he was first received, the skin was of a yellowish tint—eyes sunk—head inclining forward—some infiltration in the abdomen and lower extremities—sense of oppression in the chest, with difficulty of breathing—dull sound over a considerable extent of this quarter—pulse rather quicker than natural, but hard, full, and strong—inability to lie in any other posture than on the back—sleep very disturbed. This still continued, with some trifling variations, till the last; but never prevented the patient from getting up in the day time—even on the day of his decease. The treatment was chiefly directed towards the affection of the chest, which was considered as the principal seat of disease. With this view, general bleeding was several times practised, as well as leechings and blisterings. These produced some relief for a time only. Debility gradually increased—but the state of the pulse above-mentioned never altered, which led to the prognosis that there was inflammation of the sanguiferous vessels, and that the patient would be lost.

*Dissection.* The chest sounded dull in almost all parts of its circumference. The dura mater was slightly injected, and a considerable portion of the arachnoid was opake. No other alteration in any part of the brain. The coverings of the spinal marrow presented unequivocal marks of intense inflammation. Some portions of the medulla spinalis were softened, and there was considerable serous effusion between it and the membranes. The cellular tissue enveloping the large nervous trunks, as they issued from the spine, was inflamed, but the neurilema itself, and the medullary structure were apparently sound. In the chest there were about two pints (French) of serous effusion—lungs studded with tubercles (some of which were suppurated) and gorged with blood —larynx, trachea, and bronchia inflamed. There was considerable effusion in the pericardium—cavities of the heart filled with black blood —lining membrane of the chambers of an intensely red colour, which extended into the great vessels. The aorta and its branches, as far even as the popliteal artery, were inflamed. The carotids presented the same appearance, and the arteries of the upper extremities, near to the wrist, shewed similar phenomena. The corresponding veins were equally red and inflamed. The vessels of the short circulation presented the same appearances as those of the general circulation. Our author did not conclude that the red colour was proof of phlogosis till after numerous fruitless attempts had been made to obliterate it by washing.* In the mucous membrane of the stomach and bowels were many traces of chronic and acute inflammation. Large ulcerations and erosions were found in the neighbourhood of the ileo-cæcal valve. The liver was enlarged, and greatly degenerated in structure. The various other traces of disease we shall pass over, as of inferior importance.

*Remarks.* The first thing in this case which our author notices is the fact

---

* Our readers will see in this number some important observations on the fallacy of post-mortem appearances in the vascular system especially.

of the pulse never having been reduced by the numerous bleedings which were practised. This symptom, he thinks, will one day be acknowledged, in conjunction at least with others, as characteristic of inflammation of the blood-vessels. But as in this disease there are generally a great many other lesions going forward, the diagnosis will be always difficult. The softening of the spinal marrow in this case was a very unexpected occurrence, as the patient walked about the ward, even on the very day of his death! There was no paralysis—and the debility of muscle was not greater than might have been expected in a chronic disease of the same standing. This is one of the mysteries of the nervous system, which it would be difficult to unveil.

---

### 20. ON PURGATION, AS A REMEDY IN DISEASES.*

The intercourse between nations, like that between individuals, tends to the mutual advantage of both parties, by rubbing off the rust from either. Men are much more keen in detecting the errors of others than of themselves—and seldom very backward in portraying them to the world. In this way the Continental and British practitioners have been, for many years past, keeping up a kind of critical warfare respecting purgation, as a remedial agent, in many diseases, and especially in febrile affections. In England, since the first appearance of Dr. Hamilton's book, the sale of purgative medicines has been quadrupled, and hypercatharsis has long been the order of the day. In France, and in many other parts of the continent, the doctrines of Broussais have produced a kind of cathairo-phobia, or universal dread of purgation, in consequence of the real or supposed inflammation of the mucous membrane in many diseases, and the morbid appearances which that tissue so often presents after death. Every unprejudiced and attentive observer must see that the two extremes into which men have run, in this respect, on the north and south sides of the English channel, were attended with positive mischief in practice. The free intercourse between the two countries is now beginning to correct the errors of both. The increased and increasing attention to the pathology of the mucous membranes, in the British Isles, is checking considerably the mania for unbounded purgation here; and the good effects which the English have so long attributed to this measure, are opening the eyes of the French to the " utility and administration of purgative medicines." A late edition of Dr. Hamilton's work has just been translated into French, by M. Lafisse, and has given rise to many a hearty laugh at John Bull's *cathairo-mania*, in the French Journals and medical coteries. In a very sensible and favourable review of this translation, [Revue Medicale for December] the reviewer observes :—" The work of Dr. Hamilton has run through seven editions in the course of a few years, in England. Is the translation of the work likely to meet with similar

---

* Dr. Hamilton's Observations on Purgative Medicines in several Diseases. Eighth Edition, Ed. 1826.

success in France? assuredly not. Will this want of success prove that the work is worth nothing? No, doubtless. It will only prove that the theories of the English and French schools are very different. How, in fact, can a work which recommends the employment, nay the *abuse* of purgatives, in cases where the majority of French physicians hold these medicines to be most destructive, hope for a patient perusal; at a time too, when the aversion to purgatives is often carried to the most ridiculous extremes?"

In the course of the critique, the French reviewer, (M. Tavernier) makes many judicious reflections on Dr. Hamilton's work; and he fairly admits that the translation of it into the French language, will have the good effect of awakening the attention of continental physicians to the utility of moderate purgation in many diseases, from the treatment of which, this measure is now banished by a blind and unreasonable prejudice. We cannot help quoting the concluding passage of the review, as it may afford some entertainment to our readers.

"Ah! if French physicians should adopt the practice of Dr. Hamilton, what a number of people will consider it as the regeneration of medicine in this country! With what exultation will the whole class of pharmacopolists view the termination of a calamitous revolution, by which their shops have been left deserted, and their drugs allowed to rot! If purgation should come into vogue, what will become of the poor leeches?"

We must now dedicate a few pages to this last edition of Dr. Hamilton's work, in which the author has introduced a new chapter "on cold-bathing, considered in its purgative effect, in fever, dyspepsia, cholera, tetanus, and in the treatment of infants."

Our author sets out with questioning the truth of the general opinion, "that the efficacy of cold-bathing proceeds exclusively from its tonic or bracing powers." "Till cases," says he, "of direct debility are ascertained to exist, and to which this tonic power is applicable, and while other circumstances readily explain the influence of the cold-bath in the cure and prevention of some diseases, it will not be unreasonable to suspend our assent to a supposition which, in its narrow and limited view, sanctions the indiscriminate view of cold-bathing."

"An occasional interchange of their functions is a consequence of the connexion which subsists among the excretory organs of the body," page 11. To this interchange it is owing that different applications to the surface move the belly. Among these, that of cold water is conspicuous, whether it be made by sponging, affusion, or by resort to the cold-bath.

"Pathological facts, and the experience of those who, enjoying good health, bathe for amusement, prove the alvine evacuation to be promoted by the aspersion of, or by immersion into cold water. This effect, how greatly soever it merits attention, and however much it ought to regulate the practice of cold-bathing, both in health and in disease, has been, in a great measure, if not altogether, overlooked by late writers on the popular and interesting subject of the application of cold water to the surface of the body." 160.

To this obvious effect on the alimentary canal, the benefit derived in fever (Dr. H. thinks) from sponging with, or from the affusion of cold water, may, in part at least, be attributed. The influence of a favourite theory over the best regulated mind, will be easily seen from the following passage, in which there is a mixture of fact and hypothesis. The *latter* we cannot help considering as greatly overstrained.

"An agreeable sensation of coolness, a lowering of the pulse, a disposition to sleep, and to perspire, are the immediate consequences. Abatement of sickness, of anxiety, and thirst, and cleaning of the loaded tongue, are the subsequent effects of the impression made on the surface. By this the peristaltic movement of the intestines, retarded or suspended during the febrile state is excited, and accumulated, and noxious fæces are propelled and evacuated. The repetition of the sponging or affusion, as is generally practised, promotes the further expulsion of fæces; at any rate it will co-operate with mild purgatives, and secure daily a moderate alvine evacuation, so necessary at all times to the well conducting the cure of fever." 160.

In this passage Dr. H. evidently underrates the influence of cold on the surface, as affecting the nervous system generally, and lowering the excitement of the heart and blood-vessels. Our own observations would lead us to attribute extremely little influence to the sponging as a purgative in fever. Indeed we have rarely witnessed any decided effect of this kind on the intestinal canal, although we are ready to grant the intimate sympathy which exists between the mucous membranes and the skin.

In respect to dyspepsia, our author differs (and with good reason) from Dr. Cullen, who looked upon the disease as depending on—"imbecility, loss of tone, and weakness of the muscular fibres of the stomach." These three states of the muscular fibres are, in fact, all the same—for what is imbecility but weakness—what is weakness but loss of tone? And yet we do contend that this is not at all the proximate cause of dyspepsia, as Dr. Cullen would have discovered, had he ever laboured under the disease. It is far too mechanical a proximate cause. The nervous power, and the state of the gastric juice, (so much dependent on the nerves) must be taken into account. The muscular fibres of the stomach can only be instrumental in moving forward the layers or strata of digested matters towards the pylorus, and finally discharging the chyme through that portal. They can have no influence on the digestion itself. We agree, however, with Dr. Hamilton, that this doctrine of muscular debility in the stomach has had a pernicious influence on medical practice, as leading to the routine of "tonic medicines, bitters, astringents, and chalybeates," as well as antispasmodics and opiates. We agree with him also, in the opinion that these medicines, so indiscriminately employed, "often disappoint expectation, and by protracted use aggravate the *supposed* loss of tone, in place of remedying it." Dr. Hamilton, of course, in consonance with his favourite theory, attributes the good effects of the cold-bath in dyspepsia, to its power of opening the bowels.

"Among other tonics, operating, as it is supposed, upon the general system, and through it upon the weakened muscular fibres of the stomach, the cold-bath is mentioned. Much dependence is placed upon it, and justly so; but not, in my opinion, on account of its more than doubtful tonic powers, but on account of the impression made on the surface being communicated to the bowels, by which their languid action is excited, with a propensity to evacuate their retained contents." 163.

Whatever may be the *modus operandi* of the cold-bath, and more especially the shower-bath, the fact of its utility in dyspepsia is unquestionable; but we would beg leave to observe that there are stages and states of dyspepsia, in which the shock of the cold bath would be dangerous, and where the tepid bath is of far greater utility. The improvement of function in the stomach and bowels, through sympathy with the skin, admits of explanation without recourse to the purgative effects of cold applied to the surface.

Without admitting fully Dr. Hamilton's theory, we agree with him practically, respecting the advantages derived from sponging regularly with cold water in tropical and other hot climates. This practice tends to counteract the deleterious effects of a high temperature on the human frame. But Dr. Hamilton is quite mistaken when he supposes that the bowels become constipated in proportion as the perspiratory process is increased when we first enter a tropical climate. The case is just the reverse. So much for the speculations of the closet.

Dr. Hamilton enters his caveat against the practice of plunging infants into cold water with the view of rendering them hardy. He observes that it requires only moderately warm cloathing, a sufficient supply of light nourishing food, and free access at all times to pure air, that the child may continue in health and grow in strength. If by bathing, the important process of *ablution* only be kept in view, "it will be accomplished more effectually and safely by employing warm water, in which the child delights, and in which he loves to linger." In this sentence we are not inclined to agree with our author. We would prefer the mode, which we have often adopted and seen adopted, of first sponging or immersing with warm water, and then with cold. The prior application of the warm water brings the blood to the superficial capillaries, and enables the individual to resist the shock of the cold application infinitely better than if no previous heat be employed. One of the most important objects of cold-bathing or sponging, is the insusceptibility to atmospheric impressions which results from the practice. The insusceptibility is more certainly secured, and to a still greater amount, if the application of heat be previously made. The value of such a state of constitution in this climate will be readily appreciated. We know many people who preserve themselves from colds and coughs by this piece of hygiene. Among others, we may mention Sir Richard Phillips, a well-known character, who has not eaten any thing that ever possessed animal life for forty years past. He attributes in a great degree, (we had it from his own mouth a few weeks ago) the excellence of his health and his complete immunity from colds, to the daily ablution of his whole body with *cold water*, the moment he gets out of a *warm bed*. By this practice his skin is kept in a state of unusual purity, and its functions in good order. In consequence of the extensive chain of sympathies between the cutaneous surface and the interior organs, his health is preserved, and he is rendered entirely insusceptible of catarrhal, rheumatic, and other affections that usually result from atmospherical vicissitudes.

Dr. Hamilton has properly adverted to the utility of raising the temperature of the body to a comfortable degree of warmth, by exercise, previous to the cold bath. We strongly advise those who are subject to face-aches, coryza, &c. to get into the habit of sponging the face and neck every morning with warm water, and then immediately with cold, as a powerful preservative from these complaints.

We do not deem it necessary to notice any other part of the present edition of Dr. Hamilton's work, as the book is universally known to, and read by, British practitioners. Although we do not go the full length of Dr. H.'s views respecting purgation, we have no doubt of the general utility of the measure, under the guidance of a judicious practitioner. It is not the use but the abuse of purgatives that is to be deprecated.

---

### 21. EPILEPSY.

Mr. Scott, of Liverpool, has recently published two cases of this disease, one occurring in his own practice, the other in that of Dr. Briggs, both tending to prove the occasional benefit which may be derived from digitalis. His own patient was an untoward sample of mortality, being a boy nine years of age, and who had been nearly idiotic almost from birth. The head presented an unnatural form and appearance, and he had been epileptic for some years. The paroxysms were sometimes very frequent—many in the same day. By the long-continued administration of tincture of digitalis, which was occasionally carried to a considerable height, the boy became much better, the fits being rendered greatly milder and less frequent. At the end of the history, however, we find the youth by no means cured, nor is it very likely that he ever will, the cause of the disease being most likely organic, and within the cranium.

A long and very unconnected case is next related from the dispensary practice of Dr. Briggs; but the nature of the disease in this case is very uncertain, and the effects of digitalis too "magical," for one to place much reliance on the facts of the case, though we believe them to be faithfully stated by Dr. Briggs. The patient was 18 years of age, who, for more than three years, had been suffering from hourly attacks of what were termed "swimmings in the head," attended with violent palpitation of the heart. His countenance appeared bloated, and he sat constantly in bed, for fear of being hurt by falling. Dr. Briggs merely ordered him a common digitalis pill, containing ¼ of a grain, to be taken four times a day. By this medicine he was much relieved, and in course of time, by perseverance and the aid of some other remedies, he was able to take to his father's trade, that of a tailor. As we said at the beginning, the nature of the disease was far from obvious. Dr. B. is inclined to view the cause as more in the head than in the heart:—we should feel disposed to take the opposite view of the chain of causation, and refer the "swimming of the head" to the inordinate action of the heart. Doctors, however, will differ.—*Ed. Journ. Jan.* 1827.

In the same journal, Mr. Gunn has related the particulars of a case of epilepsy cured by purgatives. The patient was an athletic lad, 20 years of age, who was seized on the night of March 22d, while asleep, with a violent "epileptic fit," which continued severe for more than an hour. He remained in a state of stupor for a considerable time afterwards, for which he was bled and blistered. On the 25th, in the morning, the convulsion returned, and when Mr. Gunn arrived he found the patient lying comatose, with symptoms of accumulation of blood in the head—the breathing laborious—pulse 96, and full—eyes and face red—temporal arteries throbbing—tongue covered with a black crust—skin hot. Here we would ask Mr. Gunn whether this be the state which generally succeeds an epileptic seizure? The case was evidently one of *convulsions* from intestinal irritation, as will presently be shown, but it is improperly designated epilepsy.

He was now bled to 24 ounces, and another blister applied. A scruple of extract of colocynth and ten grains of calomel were administered—and calomel powders left to be taken every hour till the bowels should be well cleared. 26th. Has taken six calomel powders, in addition to the pills of colocynth and calomel—in all forty grains of calomel, with only the effect of one full alvine evacuation, "partly lumpy and particularly fetid." He is now tranquil, pulse 80—complete command of all his limbs. The tongue is furred, and he has a bad taste in his mouth. Was ordered 30 grains of the compound colocynth pill. Has had three pitchy stools. 27th. Had another paroxysm this morning, while asleep. He was ordered more purgative medicine. Mouth sore. 28th. Complains only of debility. Has had five pitchy stools. 29th. Two fetid evacuations, lumpy and dark. Had another violent convulsion last night. The purgation was continued, and he had only one more convulsion.

We think our readers will agree with us that these paroxysms were mere convulsions from the state of the bowels, and undeserving the name of epilepsy, which is not so easily cured as Mr. Gunn's case would induce the young practitioner to believe. The case, however, is valuable, as shewing well the influence of intestinal irritation on the sensorium and its functions.

### 22. EXPERIMENTS ON DIGESTION.

In one of the American Journals of Medicine (the book has been overlaid, and we take the account from a continental journal into which it is copied*) there are some curious experiments, made on the stomach of a young Canadian, who was wounded in the abdomen by the discharge of a musket, and in whom a fistulous communication between the stomach and skin existed afterwards. We shall pass over the particulars of the wound, and only state that the fistulous opening in the stomach had now continued for more than two years—that the opening was the size of a twenty-four sous piece—that the aliment taken into the stomach quickly escaped by this fistula, unless it was kept well defended by pad and compress—that the health of the young man was excellent, the appetite good, the digestion perfect, the body active, and the individual capable of every species of labour and exercise. He could, at pleasure, disgorge the aliment from his stomach, by merely removing the pad and obturateur by which the fistula was closed.

Mr. Lovell, an army surgeon, of the United States' service, had attended the patient through the cure of his wound, and then made a series of experiments on his digestion.

"When," says Mr. L. "I removed the pad, it often happened that the internal membrane of the stomach was protruded in the form of a half-blown rose, and resembling that in colour. The patient (if patient he could be called) was able to reduce this herniary protrusion by slight pressure, and without any pain. When San Martin (the young man's name) was lying on his right side, I could see into his stomach, perceive the movements of that organ, and follow, to a certain point, the different stages of digestion. I sometimes introduced water into his stomach, by means of a funnel, and solid food by means of a spoon. Afterwards I could draw them out by the aid of a syphon. I was also able to introduce pieces of meat, raw or dressed, as well as other species of aliment, tied by threads, the ends of which were kept outside. By these means I was enabled to make many curious experiments on the time necessary for the digestion of various nutritive substances. One time I introduced as a plug, or obturateur, a piece of raw beef. At the end of five hours, all that part which had gone inside of the stomach, was completely digested, and had disappeared. The division was as smooth as if it had been cut off by a knife. Every second or third day, a wine-glassful of gastric juice could be drawn off from the stomach, without any injury or pain to San Martin."

*Exper.* 1. On the 1st. of August, at 12 o'clock in the day, we introduced into San Martin's stomach, the following substances, made fast by threads: viz. a piece of alamode-beef strongly spiced—a piece

* We may here remark, that it is particularly useful as well as proper, to give accurate references, as to sources of information. We have to complain of our continental brethren, in this respect, especially the French, who are extremely negligent as to reference.

of raw corned beef—a piece of fat bacon—a piece of fresh raw beef—ditto boiled—a piece of bread—a piece of raw cabbage stalk. Each article weighed about two drachms. After they were introduced into the stomach, San Martin continued his domestic occupations, as usual, within doors. In one hour after the introduction of the above materials, they proceeded to examine them. The cabbage stalk and the bread were about half-digested—the animal food had undergone no sensible change. All the articles were forthwith returned into the stomach. At the end of two hours, the cabbage, bread, and bacon were entirely digested—the other aliments were little altered. At the expiration of three hours, the alamode beef was partly digested—the raw and corned beef was a little macerated on the surface, but its texture was nearly entire. The fluids of the stomach were somewhat rancid to the taste and smell. San Martin complained of some sense of uneasiness in the chest. At five o'clock he suffered much in his stomach—experienced a lassitude and general weakness, with head-ache. On drawing out the two bits of beef, they were found in nearly the same state as they were two hours previously—but the fluids of the stomach were still more rancid and acrid. The sufferings of San Martin prevented the re-introduction of the materials. The next morning, (2d Aug.) S. M. experienced nausea, head-ache, constipation of the bowels, depressed pulse, dry skin, coated tongue. The internal surface of the stomach, so far as it was visible, presented many white spots, like portions of coagulable lymph. Purgation was deemed necessary, and six calomel pills, of four grains each, were introduced through the fistula. In three hours, they produced a full cathartic effect, and all the symptoms were dissipated, as well as the white specks in the stomach.

*Exper.* 2. On the 7th of August, at 11 o'clock in the forenoon, the tube of a thermometer was introduced into the stomach. In five minutes the mercury stood at 100° of Fah. and continued at that standard. By means of a gum-elastic syphon, one ounce of clear gastric juice was drawn off, into a phial capable of holding three ounces. A small piece of boiled beef was immersed in the fluid. The bottle was well corked, and placed in a temperature of 100°. In about 40 minutes the digestion had evidently commenced on the surface of the meat. At 50 minutes, the fluid in the phial became opake and cloudy. The fibres of the meat began to be disengaged, and in one hour chyme seemed to be forming. At 1, p. m. the muscular fibres had diminished one half. At 5 o'clock, very few remained—and at 7, there was scarcely any visible trace of muscle. At nine the whole substance was completely dissolved. The solution had now the appearance of whey, and shortly afterwards a precipitate resembling the meat, in colour, fell to the bottom of the phial.

*Exper.* 3. On the same day, and at the same hour, a piece of meat, exactly the same size and kind as that placed in the phial, was introduced into the stomach, with a thread attached to it. At the end of one hour, it presented nearly the same appearance as the piece in the bottle. At one o'clock, the thread came away, the meat appearing to have been entirely dissolved. The process in each case was the same, for the first hour; but afterwards, the meat was much more quickly digested in the stomach than in the phial. In both cases, the digestion commenced at the surface of the meat, and seemed stationary there for a certain time. In the phial, gentle agitation seemed to quicken the solution, by presenting new points of surface for the contact of the gastric juice.

*Exper.* 4. On the 8th of August, at 9 o'clock in the morning, an ounce and a half of gastric juice was obtained and put into a phial. Two pieces of boiled chicken were suspended in the fluid by threads, and the bottle was placed in the temperature above-mentioned. A vigilant watch was kept on the whole. The digestion followed the same phases as in the second experiment, except that it was more slow. The chicken being a denser meat than the beef, the gastric juice could not insinuate itself so readily among its fibres. In other respects, the processes were precisely the same. After the digestion of the fowl, the fluid presented the appearance of milk rather than of whey, and the subsequent precipitate was of a whiter colour than that from the beef. The solutions of both were kept well corked, and in neither was there any mark of acidity or rancidity till the 6th of September following, when the beef solution exhaled a putrid odour, while that of the chicken was free from any disagreeable odour or taste.

The young Canadian was now tired of these experiments, and departed for his native place.

These experiments are curious. They are not different from what we might naturally expect, and they shew that it is to the gastric juice we owe the mysterious process of digestion, and not to any action of the muscular fibres of the stomach. These muscles merely move forward the digested strata to the pyloric orifice, as was well shewn by the experiments of Dr. Philip in the stomachs of rabbits. The effects of indigestion were satisfactorily demonstrated, when the farrago of various substances were introduced into San Martin's stomach. This experiment affords a useful lesson to the gourmand, who mixes a dozen of different kinds of viands in his stomach, regardless of the different degrees of digestibility in different substances.

---

### 23. WHITE MUSTARD SEED.*

We imagine that the author of this brochure has over-estimated the extent to which the employment of mustard seed has been carried. It *had* a great run, we know ; but, like all other catholicons, it has nearly run its day. From numerous enquiries which we have made at the

---

* A Letter on the Medical Employment of Mustard Seed. By a Member of the Royal College of Surgeons. 8vo. pp. 31, Dec. 1826.

shops where this nostrum is vended, we understand the sale of it is not now one fifth of what it was twelve months ago. The letter before us is written rather in a flippant style, and bears intrinsic proof of having been designed for a publication of equivocal respectability. But this is no business of ours. The author sets out with the narration of the case of a young girl who, being convalescent from a fever, was advised by a "talkative doctress in the neighbourhood," to swallow a quantity of the white mustard seed, by way of rapidly restoring her strength. She took six table-spoonfuls in one day, without its producing any evacuation from the bowels. The consequence was a distension of the abdomen, flushings of the face, febrile excitement, and want of sleep. Next day she persevered to the same extent, and with similar effects. On the 3d day our author saw her, when unequivocal symptoms of cerebral and abdominal inflammation were developed. Leeches to the abdomen, ice to the head, produced a temporary relief; but rigors came on, and she expired in the evening, having passed a considerable quantity of blood by stool, along with some of the mustard seed which she had swallowed. On dissection there was found general peritonitis, with some effusion of serum. When the ileum was slit open, a small jagged and ash-coloured ulcer was found, which had penetrated the mucous and muscular coats, and laid bare the peritoneal covering to the extent of a shilling, but without perforating it. There were several ulcers about the ileo-cæcal valve, and also in the track of the colon and rectum. There were also marks of inflammation in the membranes of the brain.

Our author reasonably infers that these follicular ulcerations existed during the preceding fever—he thinks they were the cause of the fever—and that they would have healed had not the irritation of the mustard seed re-kindled the constitutional disturbance and local inflammation. Several other cases came to our author's notice or hearing, where bad effects resulted from the use of the remedy. But still, as the praises of the white mustard seed rang perpetually in his ears, and as he had been more than once reminded in practice of "his own unmeritorious exertions," which had been pushed aside to make way for the favoured nostrum, he determined "*to dive into its very essence*," and set the question of its merits at rest—at least in his own mind. Our author very properly commenced on his own person, he being then in a dyspeptic state, and consequently a fair subject for a trial of the panacea. With this view he did take to it—"at first kindly and quietly swallowing the seed whole, and in faithful compliance with the prescribed regulations." He disregarded the inconvenience which it produced, during the penance of a full three weeks, although it sadly aggravated all his dyspeptic symptoms which it promised to remove, "with an unvarying inclination rather to constipate, than unbind the bowels."

"It created generally (but more particularly as the dose was increased) that sensation between pain and pleasure, which arises from an over-abundant dinner; a feeling of dryness and heat along the intestinal canal, especially about the rectum, giving also to its contents the most abominable fetor. And this odious property, I am convinced, in part, transpires through the cutaneous exhalents, as my person became not only offensive to others, but perceptibly so to myself." 15.

Our author owns that he should have slackened in his pace, even to a halt, had he not been led on by the assertion of an "*eminent physician*" that the white mustard seed was, in its operation, "*uniformly laxative.*"* Our author now gradually augmented his daily potation of this "delectable trash," till finding the "stock of his amiability decline, even to the loss of temper," he made one desperate effort, "and engorged at a meal as much as sufficed him for breakfast and dinner." In proportion as the bulk was increased, "the flatulence, costiveness, and oppression were multiplied," and, at last, to such an unspeakable extent that had it not been for the assistance of a friendly seidlitz, he might have had good cause to rue the crusade in which he had embarked.

"If not the most painful, this was by far the most disagreeable experiment to which I ever submitted. The more than putrid disgust with which I was enveloped by the disengagement of sulphuretted hydrogen gas, not only rendered me entirely miserable, but personally loathsome to all who came within reach of its unsavoury influence. Any one fond of Mustard Seed for breakfast, may take a pound or more, and he will speedily be convinced that, instead of active purgation, the *faculty of distentiveness* is its chief characteristic." 17.

From personal and other experience then our author comes to the conclusion, that white mustard seed swallowed whole, or taken in any other way, "possesses no intrinsic property to make it worth the notice of the profession, or the invalid." The author censures Dr. Paris for borrowing the authority of M. Julia Fontenelle, respecting the *virtues* of white mustard seed, instead of writing from "actual observation." On this point we say nothing; but we cannot help animadverting not only on the severity but on the *error* of the following passage.

"This learned and distinguished Physician has lately put forth an

---

* Against whom the following pungent note is levelled, we shall not pretend to divine, though it might not require the assistance of a witch to guess.

"The pitiable labours of the would-be author, are a continual disgrace to English medical literature. I mean of one whose only talent consists in dressing up in a new language the property of others, and then palming the miserable pilferings upon his brotherhood, as the fruits of his own industry. Let a student purchase half a dozen writers who have treated the same disease (Dyspepsia for instance), and he will find the greater part of each work occupied by the same anatomical and physiological history of digestion, and on coming to the point of a thick 8vo. ten or twelve shilling volume, the useful matter is easily condensible within a half-sheet of foolscap.—I suppose, in the present crowded state of the profession, it is really necessary for a man to write something—any thing—before he can emerge from his natural obscurity:—this is certain, that in these days of empiricism, he who deals most in the marvellous, is the most likely to make his fortune."

elementary work on *Peptic Philosophy.* At page 172 we read, that new bread is indigestible, and unwholesome, because 'it swells in the stomach like a sponge.'—Now, if I had a pupil in physiology of three months' standing who betrayed such a woful lack of information as this, I should blush exceedingly for his tutor. It is impossible that *any thing* can *swell* in the stomach.—The distention, here unwittingly referred to the dilatation of the substance itself, is occasioned by the disengagement of carbonic acid gas, the bread being ill fermented."

On what authority does the author rest his unqualified assertion that nothing can swell in the stomach? Why should not new or any other bread swell in the stomach as well as in a bason of warm water into which it may be thrown? Every man's own sensations assure him that alimentary substances do swell in the stomach, independently of the extrication of gas or air. But if he consults the experiments of Spalanzani, and especially those of M. Gosse, of Geneva, he will find the observation of Dr. Paris correct.

In respect to the white mustard seed, we have not given it much trial, either on ourselves or our patients; but from the assertions of men, on whose testimony we think we could rely, it has occasionally proved useful as an *aperient*, which quality it certainly does possess, though by no means in a manner that can be relied on in practice. On the other hand, we have seen four or five instances where this nostrum caused considerable disturbance in the system that required medicinal aid, and made work for the doctor. On the whole, we apprehend that the white mustard seed, like most other popular remedies, employed by the patient himself, will not affect the art and mystery of the regular practitioner; it will do at least as much harm as good—if not more. The greater the sale therefore of popular medicines and popular medical works, the more grist for the professional mill. If the public will be gulled—let them!

Qui vult decipi decipiatur.

---

### 24. ALTERATIONS IN THE STATE OF THE BILE.

In this country, the condition of the secretions, and especially of the biliary secretion, has long attracted attention. But, on the Continent, physiologists and pathologists seem to have been too exclusively employed, till of late, in ascertaining the condition of the solids, rather than the fluids of the body. We are glad to see such men as Andral taking up the enquiry. In a late sitting of the Parisian Academy of Medicine, this observant pathologist read a memoir on the alterations of the bile, which we hope will soon be published. In the mean time, we can only state a brief outline of the paper. M. Andral shewed that we have often bile apparently healthy, when the liver is in a state of disease; and that, on the other hand, this secretion is often extremely vitiated, without any perceptible lesion of structure in the organ which produces it. One species of alteration which he has frequently observed, consists in a thin, aqueous, and albuminous condition of the bile, with very little yellow colour. He has seen this alteration in fatty degeneration of the liver—in atrophy of this organ—in hypertrophy—induration—and scirrhus. He has also seen it in diseases where the liver was not affected apparently. He has seen the bile (as every one may see, who attentively observes) varying in colour, from a bright yellow to a jet black—in consistence, from the clearness of water to the density of honey. He has seen some specimens of bile that has blistered the skin when applied thereto—and, when introduced into the cellular membrane of an animal, it has proved a veritable poison. These various alterations of the bile, he thinks, must justify the general opinion of the English, that the state of this fluid plays an important part in many derangements of the digestive organs. The physicians of England, says he, endeavour to remedy these biliary derangements by purgation, and especially by calomel and blue pill; whereas the disciples of Broussais consider these derangements as more dependent on chronic gastro-enteritis than on vitiation of the bile, and, consequently, proscribe purgation from the list of their remedial agents. Which party are we to believe? M. Andral thinks that both have carried their doctrines too far—and that both are sometimes right, as well as sometimes wrong. As for himself, he has found purgatives very useful in many cases, where Broussais would have condemned them as poisons.

M. Andral has made many observations on icterus. Every organic disease of the liver may produce the yellow colour of jaundice—and all these diseases may exist without any icteritious suffusion. In general, where jaundice has been seen, without any organic lesion or mechanical obstruction in the liver, he has discovered the existence of either duodenitis, inflammation of the diaphragmatic pleura of the right side, or some phlegmasia of the brain or its coverings. In a few cases, however, he has been able to discover no organic lesion in any part of the body. In these instances, he thinks the cause of jaundice must have been some powerful mental emotion, or some affection of the hepatic plexus of nerves.

In respect to the yellow colour of the skin in jaundice, physiologists in general attribute the phenomenon to resorption of the bile, and its transport into the current of the circulation. M. Andral is rather disposed to think that the icteritious tint is produced by non-secretion, and, consequently, the remora in the blood, of the elements of bile, than by resorption of it after it it is secreted in the liver. On this point we must differ, toto cœlo, from M. Andral. We know that the accidental entanglement of a gall-stone in the ductus communis will, even in a few hours, render the skin yellow. How can this be attributed to non-secretion? In those individuals, and in those complaints, where there is evidently a great paucity of bile, we find quite a different kind of complexion from jaundice—namely, a sallowness, which it is difficult to describe. We grant, however, that the yellow colour of the skin in yellow fever—in new-born infants—and after bruises, is very difficult to account for, either by the theory of non-secretion or resorption. We shall look with anxiety for M. Andral's memoir on this subject in a larger shape.—*Archives.*

### 25. SECALE CORNUTUM. MR. HASLAM.

Mr. T. B. Haslam, of Caernarvon, has lately administered this substance, with success, in cases of lingering labour, and also of uterine hæmorrhage. The following will serve as examples.

*Case* 1. Mrs. —— had been in labour fifteen hours before Mr. H. saw her. The pains were weak, the membranes ruptured, os uteri dilated, thin, and yielding. One scruple of powdered ergot of rye was given in thin gruel. In less than an hour strong pains came on, and by the expiration of the second hour the patient was safely delivered.

*Case* 2. A lady had been in labour 23 hours before Mr. H. was called. She stated that the pains were very weak, and now flagging altogether; the membranes are entire; os uteri not much dilated, and rather rigid. This was the lady's first labour, and Mr. H. waited eight hours, without any advancement or increase of the pain. One scruple of the ergot was, therefore, given, the os tincæ being dilatable. In three quarters of an hour strong pains came on, and safe delivery ensued. Mr. H. is of opinion that, in these two cases, delivery would not have been effected, without instrumental aid, had not the ergot been administered.

*Case* 3. The patient had been in labour 18 hours, during four of which the head was far advanced, but not continuing to advance. The patient's strength was failing. Under these circumstances, Mr. Haslam administered the ergot, which excited powerful pains, and the child was born in an hour and a quarter.

Mr. H. has given the ergot in cases of uterine hæmorrhage, with similar results. But, in puerperal convulsions, the ergot did not appear to excite any specific action, or answer any purpose.

The foregoing cases may encourage practitioners to farther trials of this curious medicine; and we should be glad to know the result, which we shall communicate from time to time, as upon the present occasion.

### 26. MOLLESCENCE OF THE LUNGS.

In the January number of our respected Northern contemporary, the Ed. Journal of Medical Science, Dr. Hastings, of Worcester, has described a morbid state of lungs which does not exactly correspond with any that we have met with among modern pathological investigations. This is a peculiar softening of the pulmonary texture, without any tubercular disorganization, or any of the usual effects of inflammation. In consequence of this mollescence, profuse hæmorrhages sometimes occur—sometimes large ulcerous cavities are formed, not tuberculous, nor surrounded by any hardening of the parenchyma, as in hepatization. Sometimes the cellular tissue of the lung passes into a pulpy state, and makes its way through the bronchia by expectoration, without any ulcerated cavity being formed. This pathological condition has presented itself to our author in very different states of the constitution:—sometimes in connexion with fever—sometimes with gradual failure of the vital powers, and slow emaciation—but, in most instances, without symptoms indicative of much mischief in the respiratory organ. Its tendency, in all cases, is to unfit the lungs for their office, and consequently to destroy life. Six cases in illustration are detailed by our author, of which we shall notice one or two.

*Case* 1. This was one of fever, in a man, 24 years of age, and who was not seen till the stage of collapse had taken place, with petechiæ on the surface. He seemed, however, to be recovering; but on the 27th day of his illness, there came on difficulty of breathing, &c. and he died on the 31st day of the disease.

The lungs did not collapse when the sternum was raised. They were exceedingly soft in texture, " and very much resembled the spleen when in a very soft state." In many places, there was a dark bloody fluid between the pleura pulmonalis and the parenchyma. The trachea was found full of a bloody serous fluid, as were the ramifications of the bronchia. In the air-cells the membranous appearance was lost, and only a soft pulpy matter was discoverable.

We shall be able to notice only another case, which is an interesting one.

*Case* 2. A young lady had been in a dubious state for two months, in consequence of cough, some trifling expectoration in the mornings, and occasional tinges of blood in the sputa. Three of the same family had died of consumption. The young lady, however, was " full of flesh," and was gaining rather than losing in that respect. Still she had an unhealthy aspect—the pulse about 76—the tongue clean—bowels regular—breathing free—catamenia regular. In short, she complained of nothing except a dry hacking cough on first getting up in the morning. On percussion, the chest emitted a natural sound on each side, and the respiration was heard over both sides. Perfect pectoriloquism was heard on the left side, between the second and third, and third and fourth ribs. Our author therefore concluded, that there was a cavity in the superior lobe of the left lung. It was considered to be of small extent, and therefore recovery possible—and, at all events, danger not imminent. Cupping between the shoulders was prescribed, and infusion of roses with tincture of digitalis every four hours. Mild unirritating diet. In a few days the blood disappeared from the expectoration, and the young lady considered herself as " in perfect health," except a trifling cough. Another examination with the stethoscope confirmed the former diagnosis of an excavation in the lungs. She went on for about a month pretty well, when one morning she brought up some blood, accompanied by a small quantity of purulent matter. Alum was added to the mixture above-mentioned, and the pil. aloes cum myrrha prescribed. The hæmoptysis proceeded no farther, and again she thought herself getting quite well. In ten days more, some hectic fever shewed itself, with some ill-looking expectoration. In three days from this period there was expuition of scarlet-coloured blood. She complained of pain between the shoulders. Quietude—alum, infusion of roses, tincture of digitalis—cupped between the shoulders. The hæmoptysis continued the next day, and a grain of superacetate of lead, with a quarter of a grain of opium, every two hours, were ordered. These means proved inefficient, and she died in a few days.

On removing the lungs, they were found slightly adherent on the right side, and more so on the left. There was no appearance of purulent secretion in the trachea. The bronchia contained some sero-purulent fluid. On tracing the ramifications in the right lobes, the only appearances were, a rather suffused state of the blood-vessels in some parts—the lungs of a soft structure—and the air-vessels pervious throughout. This state obtained in the left side; "but on the upper and outer surface of the superior lobe appeared a cavity large enough to admit the half of a middle-sized orange, which had been prevented from opening into the cavity of the chest by the adhesion of the surface of the lung to the costal pleura; but it communicated freely with the bronchial tube." Pus was searched for, but could not be found:—Nor was there the smallest degree of hardness in the sides of this ulcer, indicative of the process of adhesive inflammation having been set up. On the contrary, the appearance was that of simple ulcerative absorption.

Dr. Hastings has appended some judicious reflections to this and the other cases, for which we must refer to the original paper. We shall, however, quote one passage, as it bears upon a topic which we have once or twice introduced into this journal.

"May not we also take advantage of such cases as the above, and by them enforce the necessity of treating certain varieties of pulmonary consumption by a strictly tonic plan? And may not this plan, so much reprobated by some authors, as not according with the results of examination after death, be found consistent with, or at least not opposed to, the evidence of morbid anatomy?"

The organic changes which the lungs undergo in disease are extremely numerous, and many of them are quite insusceptible of diagnosis, by the most accurate pathologist, during life. A man lately came into St. George's Hospital, with the *left* side of the thorax somewhat bulged out, and completely devoid of sound or respiratory murmur, on percussion and auscultation. In the *right* side of the chest, the heart was seen and felt beating; but respiration could not there be heard, except high up near the clavicle, and backwards near the spine. The respiration was laborious, the lips blue, the pulse quick. No accurate history of the complaint could be obtained. The patient stated that he had been ill six weeks, with pain in the lower part of the sternum, cough, but no expectoration. It was pretty evident that there was fluid in the left side of the thorax, and as it might possibly be purulent, it was agreed, in consultation, that an opening should be made between the sixth and seventh ribs. This was done by Mr. Brodie, when a considerable quantity of sero-sanguineous fluid issued. The patient experienced great relief, and slept for some hours uninterruptedly after the operation. He gradually sank, however, and expired in a day or two. After the operation, the sound immediately returned in the right side, where the pulsation of the heart had previously been felt. The *left* side sounded hollow, of course, from the unavoidable admission of some air during the operation. On dissection, we understood that a large fungus hæmatodes had occupied and disorganized the left lung, as well as the corresponding portion of diaphragm, descending to the spleen.

We think the physician (Dr. Hewett) who recommended, and the surgeon (Mr. Brodie) who performed the operation, were perfectly justifiable. The man was suffering great distress in breathing, and no one could tell what was the precise nature of the fluid in the chest. Its removal, therefore, was desirable, whatever might be its nature. The great relief which followed did more than compensate for the pain of the operation, which last was justifiable on the principle of Euthanasia.

---

### 27. DEATH AFTER REMOVAL OF A TESTICLE.

The fatal accidents which succeed wounds and operations, not in themselves very formidable, have recently attracted much attention, and ought to be accurately investigated, *post mortem*, as this investigation must be attended with great advantages. The post-mortem research, in the following case, is imperfect, as we shall presently shew.

A man was received, under Mr. Lawrence, with an enlarged testis, of several years' standing. Various means of reduction were employed, but in vain: and, the man being otherwise in apparent good health, the testicle was removed. It was found to be of scirrhous construction. After the operation, the patient experienced symptoms resembling enteritis, and died at the end of seven days, "without any apparent urgent cause."

*Dissection.* The wound had suppurated freely, but no adhesion had taken place. The viscera of the abdomen appeared healthy, at first view, but a portion of intestine in the pelvis was found highly vascular and red for the space of six inches, and its internal surface ulcerated. A tumour, not very dissimilar in structure to that of the diseased testis, was discovered attached to the kidney.—*Lancet, No.* 179.

The state of the lungs and liver is not specified, and no mention is made of the brain. The mere redness of the intestine, for so small a space, and the ulceration of its mucons membrane, do not afford physiological explanation of the patient's death, and we are convinced that a further and more minute investigation would have disclosed lesions in the cerebral, pulmonary, or biliary apparatus. We cannot, therefore, too often or too strongly urge our brethren to examine all the great cavities of the body, when they are seeking for the cause of death, else they will frequently deceive themselves and others.

